# Study of the intestinal microbiota composition and the effect of treatment with intensive chemotherapy in patients recovered from acute leukemia

**DOI:** 10.1038/s41598-024-56054-w

**Published:** 2024-03-07

**Authors:** Xenia Vázquez, Pilar Lumbreras-Iglesias, M. Rosario Rodicio, Javier Fernández, Teresa Bernal, Ainhoa Fernández Moreno, Paula López de Ugarriza, Ana Fernández-Verdugo, Abelardo Margolles, Carlos Sabater

**Affiliations:** 1 Traslational Microbiology Group, Instituto de Investigación Sanitaria del Principado de Asturias (ISPA), Oviedo, Spain; 2 Dairy Research Institute of Asturias (IPLA), Spanish National Research Council, (CSIC), Villaviciosa, Asturias Spain; 3 Department of Clinical Microbiology, Hospital Universitario Central de Asturias (HUCA), Oviedo, Spain; 4 Department of Functional Biology, Microbiology Area, University of Oviedo, Oviedo, Spain; 5 Research & Innovation, Artificial Intelligence and Statistical Department, Pragmatech AI Solutions, Oviedo, Spain; 6 Centro de Investigación Biomédica en Red-Enfermedades Respiratorias, Madrid, Spain; 7 Department of Hematology Instituto de Investigación Sanitaria del Principado de Asturias (ISPA), Instituto de Oncología del Principado de Asturias (IUOPA), Hospital Universitario Central de Asturias (HUCA), 33011 Oviedo, Spain; 8 Instituto de Investigación Sanitaria del Principado de Asturias (ISPA), MicroHealth Group, Oviedo, Spain

**Keywords:** Acute leukemia, Metagenomics, Microbial metabolism, Cross-feeding, Chemotherapy, l-asparaginase, Metagenomics, Genome informatics, Sequence annotation, Clinical microbiology

## Abstract

A dataset comprising metagenomes of outpatients (n = 28) with acute leukemia (AL) and healthy controls (n = 14) was analysed to investigate the associations between gut microbiota composition and metabolic activity and AL. According to the results obtained, no significant differences in the microbial diversity between AL outpatients and healthy controls were found. However, significant differences in the abundance of specific microbial clades of healthy controls and AL outpatients were found. We found some differences at taxa level. The relative abundance of *Enterobacteriaceae*, *Prevotellaceae* and *Rikenellaceae* was increased in AL outpatients, while *Bacteirodaceae*, *Bifidobacteriaceae* and *Lachnospiraceae* was decreased. Interestingly, the abundances of several taxa including *Bacteroides* and *Faecalibacterium* species showed variations based on recovery time from the last cycle of chemotherapy. Functional annotation of metagenome-assembled genomes (MAGs) revealed the presence of functional domains corresponding to therapeutic enzymes including l-asparaginase in a wide range of genera including *Prevotella*, *Ruminococcus*, *Faecalibacterium*, *Alistipes*, *Akkermansia*. Metabolic network modelling revealed potential symbiotic relationships between *Veillonella parvula* and *Levyella massiliensis* and several species found in the microbiota of AL outpatients. These results may contribute to develop strategies for the recovery of microbiota composition profiles in the treatment of patients with AL.

## Introduction

Gut microbiota contains the majority of microorganisms present in humans. This microbial community is estimated to have at least the same number of bacterial cells than human cells^1,2^. Along with bacteria, other life forms also reside in the gastrointestinal tract in the minority, such as fungi, viruses, archaea and protozoa. Metabolic interactions between the gut microbiota and the human body contribute to maintain intestinal homeostasis. The predominance of several microbial phyla including *Firmicutes*, *Bacteroidetes*, *Proteobacteria*, *Actinobacteria*, *Fusobacteria*, and *Verrucomicrobia* is commonly associated with a healthy gut microbiota. In this regard, the phyla *Firmicutes* and *Bacteroidetes* usually represent the 90% of gut microbiota^3^. However, specific alterations in the microbiome can contribute to the development of certain diseases^4^.Changes in the composition of the microbiota have been associated with several types of cancer such as leukemia. Leukemia is a haematological malignancy related to the uncontrolled proliferation of mutant progenitors, suppressing the production of normal blood cells and leading to pancytopenia and high infection and bleeding risk^5–7^. In 2020, 470,000 cases of leukemia were diagnosed worldwide, representing 2.5% of cancer cases in the world^8^.

Leukemia can be classified into two groups based on growth and cell line of origin. Based on how quickly the disease progresses, there are two types of leukemia, chronic and acute. Chronic leukemia targets mostly adults and is usually overpowered at a gradual rate, whereas acute leukemia (AL) develops quickly. In addition, AL may involve lymphoid or myeloid precursors, resulting in acute lymphoblastic leukemia (ALL) or acute myeloid leukemia (AML), respectively. Whereas ALL is the most frequent type of AL of childhood and adolescence, AML is the most frequent type of AL of adults^9^. In addition, AML can be preceded by preleukemia states, what are called myelodysplastic syndromes (MDS). The risk of AML evolution for a particular MDS is calculated taking into consideration clinical and genetic variables^10^. Hence, two large groups of MDS patients-low and high-risk, can be differentiated in terms of their clinical evolution^11^.

Curative treatment of AL and high-risk MDS relies on the administration of intensive chemotherapy with or without stem cell transplant, which is applied depending on the prognostic features of the disease^12–15^. It should be noted that microbiome changes have been linked not just to leukemia-induced microenvironmental changes, but also to chemotherapy or antibiotic treatment, and may cause or exacerbate dysbiosis and infectious complications^16^. Chemotherapy, radiotherapy and immunotherapy have all been shown to detrimentally impact the composition of the gut microbiota, with changes in its composition identified years after treatment. For example, a reduction in gut microbial diversity during induction chemotherapy in acute lymphoblastic leukemia patients has been reported^17^. These changes are increased by high rates of antibiotic use, disease-associated stress, and changes in dietary habits.

On the other hand, next-generation sequencing (NGS) technologies allow a comprehensive characterisation of gut microbiota composition and functionality. In this regard, the applications of shotgun metagenomics to recover metagenome-assembled genomes (MAGs) have been reported^18,19^. Bioinformatic methods based on MAG genome annotation and metabolic models can be used to simulate metabolic interactions between different gut microbe communities. These methods could be of great interest to investigate the role of microbial metabolism in several pathologies associated with gut dysbiosis^18^. However, the majority of studies deal with the recovery of MAGs from healthy microbiota samples.

Few studies report the applications of metagenomics to characterize the microbiota of AL patients. Metagenomic analyses of bloodstream infections in patients with acute leukemia have been performed while MAGs were recovered from faecal samples of AL patients with gut colonization by Multidrug-resistant (MDR) *Enterobacteriaceae*^20,21^. Moreover, in a recent work carried out by our research group, shotgun metagenomics revealed the absence of extended-spectrum betalactamases (ESBL)- and/or carbapenemase-producing *Enterobacterales* in the gut microbiota of 28 outpatients who had recovered from AL, supporting that outpatients were truly decolonized^22^. To our knowledge, no studies report a comprehensive characterisation of microbiome composition and function of decolonized individuals, which may have important repercussions for the future clinical management of the patients. In addition, no studies report the potential of shotgun metagenomics and metabolic modelling to elucidate metabolic interactions between microbial communities in the context of AL.

Therefore, the aim of this study was to characterize the gut microbiota of 28 outpatients who have recovered from AL (AML, MDS and ALL) at taxonomic and functional level using shotgun metagenomics. Then, MAGs were recovered and annotated to elucidate metabolic activities involved in AL remission and metabolic interactions between these microbial communities were reconstructed using bioinformatic methods.

## Results

### Assembly-free analysis

#### Microbial diversity

**Figure 1:**
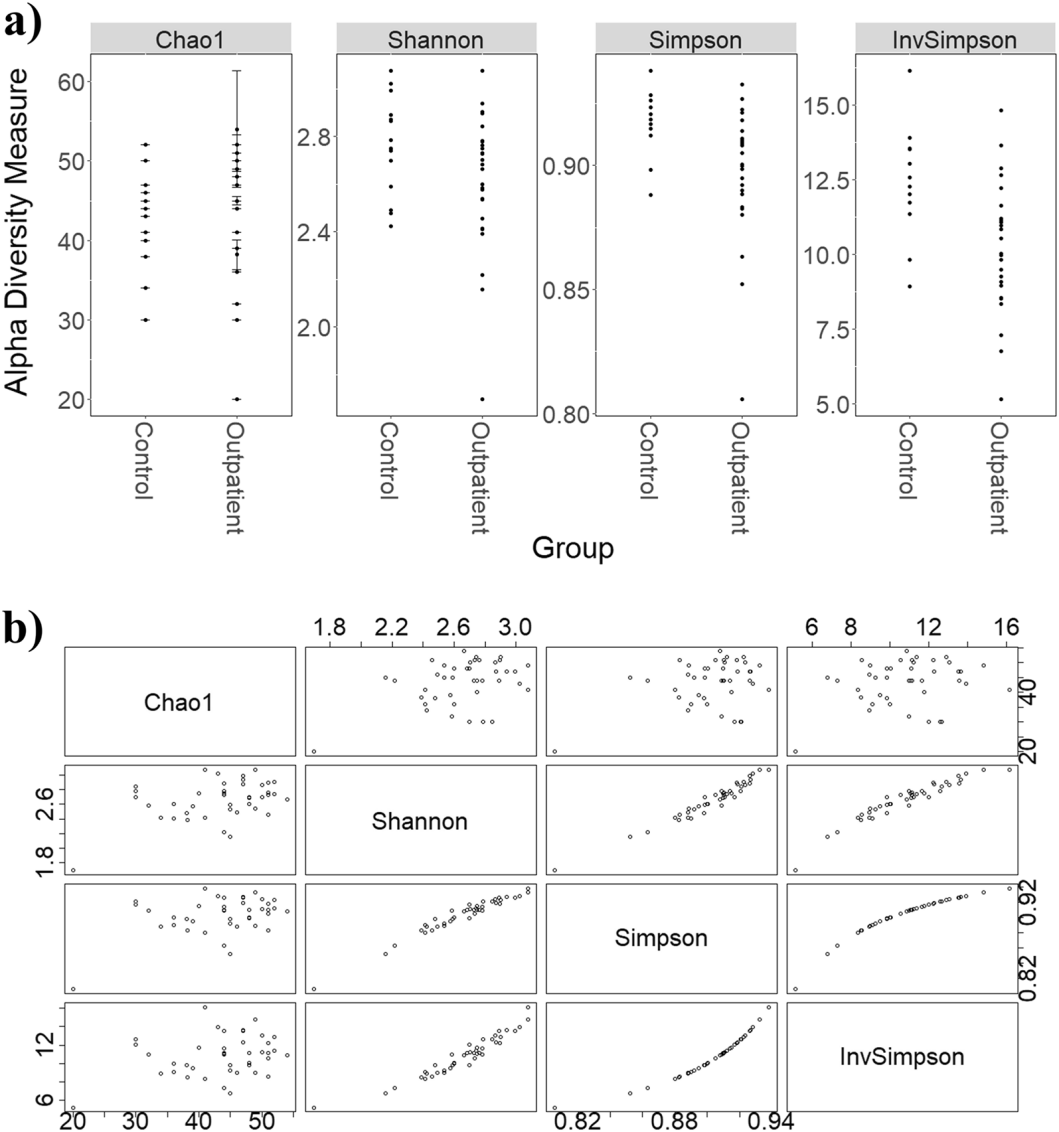
(**a**) Comparison of different alpha-diversity indicators (Chao1, Shannon, Simpson and Inverse Simpson) of the relative abundance of taxa determined at species level in outpatients recovered from acute leukemia (AL) or that underwent stem cell transplantation and healthy controls. (**b**) Relationship between different alpha-diversity estimators: Chao1, Shannon, Simpson and Inverse Simpson indices. These coefficients reflected similar patterns in the microbiota composition. ^a,b^Statistically significant (*p* < 0.05) differences between groups.

An assembly-free analysis was first performed to characterise microbial communities of outpatients of AL and healthy controls. Changes in the microbial composition of samples at both taxonomic and functional levels were determined. Concerning taxonomic analysis, alpha diversity measuring the variability of species within a sample was calculated. The alpha diversity estimators, such as Chao1, Shannon, Simpson, and inverse Simpson indices, were calculated to characterize microbial diversity at species level (Fig. 1a). Shannon, Simpson, and inverse Simpson indices reflected similar patterns in the microbiota composition within samples without relevant changes in diversity microbial, confirming the results from the different analyses (Fig. 1b). In this regard, Chao1 and Shannon indexes showed no significant differences (*p* > 0.05) among samples while Simpson and inverse Simpson estimators were significantly (*p* < 0.05) higher in healthy controls than outpatients recovered from AL, the latter showing a high intrasample variability. Then, beta diversity estimators based on Bray–Curtis distances were calculated to estimate microbial diversity between individuals within a group (Supplementary Figure S1). This parameter reflects differences in microbial diversity between samples. As can be seen, beta diversity estimators calculated for taxonomic composition data (Supplementary Figure S1a) microbial gene families and metabolic pathways (Supplementary Figure S1b, c) were significantly higher (*p* < 0.05) in outpatients recovered from AL than healthy controls. These significant differences highlight the variability between the samples of the outpatient group.

In addition, diversity distances were used to cluster metagenomes (Supplementary Figure S2). Most metagenomes of outpatients recovered from AL were clustered together although some samples corresponding to the healthy control group were clustered in the same branch as outpatient metagenomes. This behaviour was observed in clusters generated for both taxonomic (Supplementary Figure S2a) and functional data (Supplementary Figure S2b, c) and may be attributed to the high interindividual variability of microbiota of samples corresponding to each group. This variability exerts a great influence on the global metagenomic profile. With regard to the functional analysis of metagenomes, a similar gene count was determined for both groups: healthy controls (mean 9028 ± 2281; median 9817) and outpatients recovered from AL (mean 6399 ± 4127; median 7989). No statistically significant differences (*p* > 0.05) were found in the gene count of these groups.

#### Statistical analysis of the microbiota

A principal coordinates analysis (PCoA) of complete microbial taxa present in the microbiota of each group was computed to study in depth the differences in microbiota profiles of samples (Supplementary Figure S3). Regarding the taxonomic profiles, no characteristic patterns could be found for control and outpatient samples with the exception of two outpatient metagenomes that showed a high variability in their composition compared to the rest of samples from this group (Supplementary Figure S3a). However, PcoA discriminated several samples from healthy control and outpatient groups based on microbial gene families (Supplementary Figure S3b) and metabolic pathways (Supplementary Figure S3c). It should be noted that samples could not be properly discriminated due to the high intragroup variability in outpatient metagenomes, in agreement with alpha and beta-diversity analyses (Fig. 1 and Supplementary Figure S1). Nevertheless, statistically significant differences (*p* < 0.05 and *p*_*adj*_ < 0.05) in specific taxonomic clades and metabolic functions between healthy controls and outpatients recovered from AL were determined (Table 1 and Supplementary Table S3).

**Table 1:** Microbial taxa showing the highest abundances in the microbiota of outpatients recovered from acute leukemia (AL) and healthy controls.

Level	Taxa	Controls		Outpatients	
		Mean	SD	Mean	SD
Taxonomic clades showing higher abundances in healthy controls					
Phylum	*Actinobacteria*	7.76	12.18	1.89	3.93
Phylum	*Firmicutes*	34.70	15.91	24.29	15.54
Class	*Clostridia*	29.12	17.58	16.14	15.13
Order	*Bifidobacteriales*	5.84	11.65	0.97	3.77
Order	*Clostridiales*	29.12	17.58	16.14	15.13
Family	*Bacteroidaceae*	42.85	19.64	25.56	18.92
Family	*Bifidobacteriaceae*	5.84	11.65	0.97	3.77
Family	*Lachnospiraceae*	15.99	12.20	6.67	8.90
Genus	*Bacteroides*	42.85	19.64	25.56	18.92
Genus	*Bifidobacterium*	5.84	11.65	0.23	0.26
Genus	*Blautia*	8.61	9.62	0.93	2.33
Species	*Bacteroides vulgatus*	16.53	13.50	8.16	11.09
Taxonomic clades showing higher abundances in outpatients recovered from acute leukemia (AL)					
Phylum	*Proteobacteria*	3.54	5.07	13.01	23.30
Class	*Gammaproteobacteria*	2.62	4.23	11.40	23.57
Order	*Enterobacterales*	2.25	4.28	11.25	23.59
Family	*Enterobacteriaceae*	2.25	4.28	10.61	23.41
Family	*Prevotellaceae*	4.00	10.47	22.13	25.07
Family	*Rikenellaceae*	1.72	3.15	4.77	6.59
Genus	*Alistipes*	1.72	3.15	4.77	6.59
Genus	*Prevotella*	4.00	10.47	22.08	25.11

Mean abundances and standard deviations (SD) of taxonomic clades are shown.

**Figure 2:**
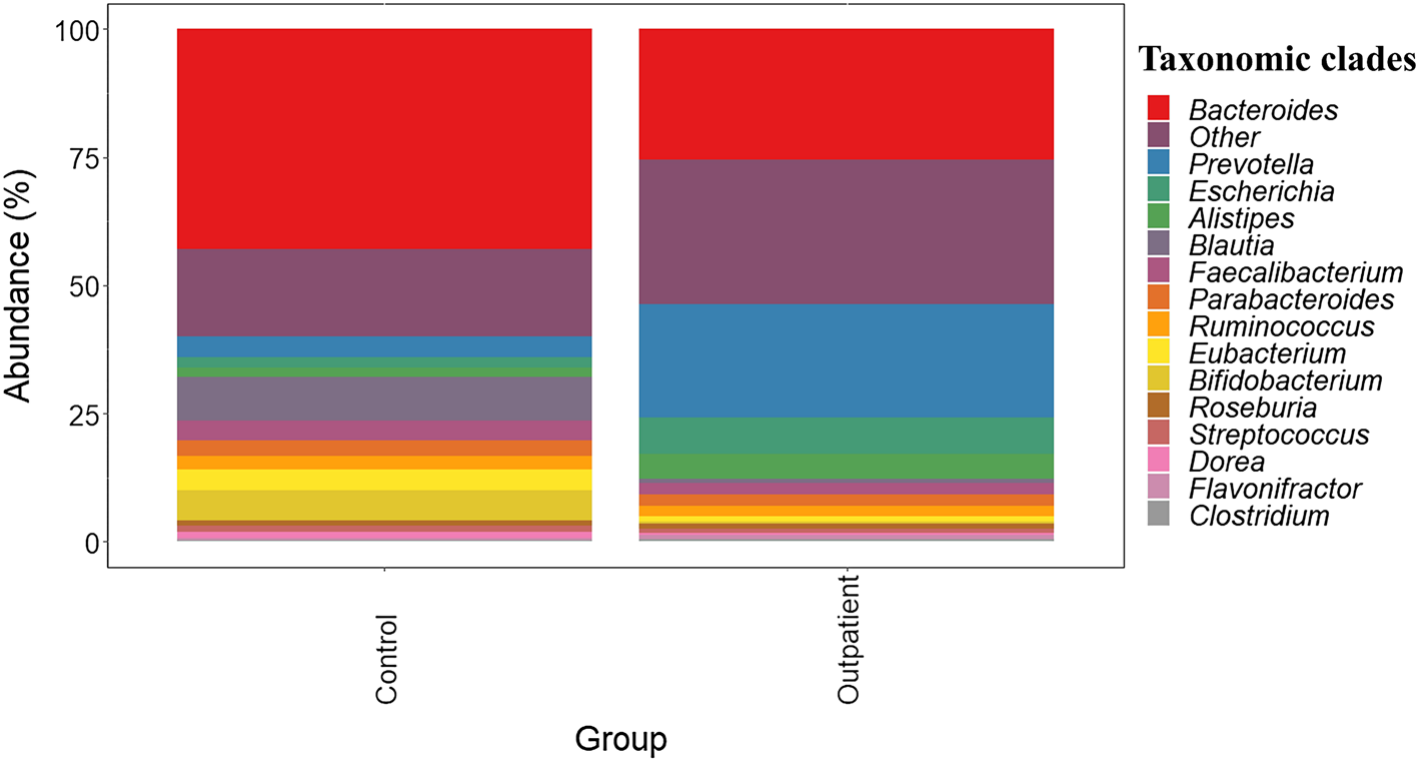
Most abundant taxa found in the microbiota of outpatients recovered from acute leukemia (AL) and healthy controls. These taxa constitute the core microbiota of individuals. Data are expressed as abundance percentages (%).

The characteristic microbiome pattern of healthy controls (Table 1, Supplementary Figure S4) comprised two main phyla (*Actinobacteria* and *Firmicutes*), two orders (*Bifidobacteriales* and *Clostridiales*), three families (*Bacteirodaceae*, *Bifidobacteriaceae* and *Lachnospiraceae*) and three genera (*Bacteroides*, *Bifidobacterium* and *Blautia*). These characteristic genera were significantly higher (*p* < 0.05 and *p*_*adj*_ < 0.05) in healthy controls than outpatients recovered from AL. The microbiome of these individuals was enriched to a lesser extent with the genera *Prevotella*, *Alistipes*, *Escherichia*, *Faecalibacterium*, *Parabacteroides*, *Ruminococcus* or *Dorea* although these differences were not significant due to intragroup variability (*p* > 0.05 and *p*_*adj*_ > 0.05). In this regard, a graphical representation of major differences in the abundances of microbial species is provided in Fig. 2.

On the other hand, the characteristic microbiome pattern of outpatients recovered from AL (Table 1) comprised *Proteobacteria* phylum, *Gammaproteobacteria* class, *Enterobacterales* order, three families (*Enterobacteriaceae, Prevotellaceae* and *Rikenellaceae*) and two genera (*Alistipes* and *Prevotella*). The abundance of these two characteristic genera was significantly higher (*p* < 0.05 and *p*_*adj*_ < 0.05) in outpatients recovered from AL than in healthy controls. A significant (*p* < 0.05 and *p*_*adj*_ < 0.05) decrease in the relative abundance of *Bacteroides*, *Blautia* and *Bifidobacterium*, three of the most abundant taxa in the microbiome of healthy controls, was detected in colonized samples. Notably, a positive correlation expressed as Pearson correlation coefficients (*p* < 0.05) was observed between chemotherapy time and the presence of *Bacteroides vulgatus*.

**Figure 3:**
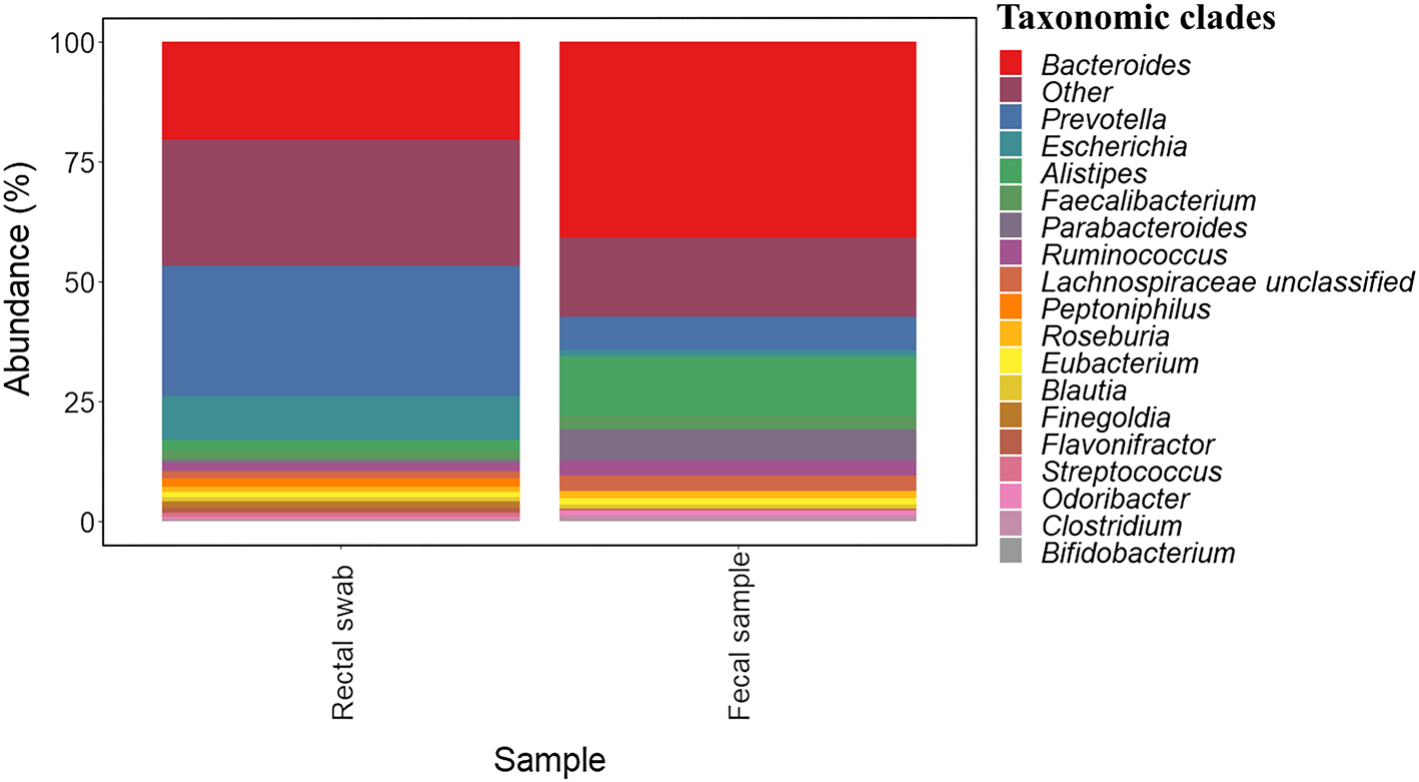
Most abundant taxa found in the microbiota of rectal swab and faecal samples of outpatients recovered from acute leukemia (AL). These taxa constitute the core microbiota of individuals. Data are expressed as abundance percentages (%).

Interestingly, no statistically significant (*p* > 0.05 and *p*_*adj*_ > 0.05) differences in the microbiota composition of male and female outpatients was observed. However, some differences were observed between rectal swabs and faecal samples (Fig. 3). *Bacteroides* and *Prevotella* were the most abundant genera in faecal samples and rectal swabs, respectively. In addition, a high abundance of *Escherichia* was found in rectal swabs, while *Alistipes*, *Parabacteroides* and *Lachnospiraceae* showed high abundances in faecal samples.

In addition, differences in outpatient recovery time from the last cycle of chemotherapy to sampling were investigated. Samples were grouped into outpatients with early recovery or T1 (< 2 years) and outpatients with delayed recovery or T2 (≥ 2 years), but no significant differences were detected. However, small differences were observed at the taxonomic level (Supplementary Figure S5). The species *Prevotella buccallis* and *Eubacterium rectale* were most abundant in T1 outpatients, while a high abundance of *Escherichia coli*, *Bacteroides uniformis*, *Faecalibacterium prausnitzii* and *Bacteroides ovatus* was found in T2 outpatients compared to T1 outpatients.

With regard to significant (*p* < 0.05 and *p*_*adj*_ < 0.05) differences found in functional profiles, up to 1565 and 58 characteristic microbial gene families were determined for healthy controls and outpatients recovered from AL (Supplementary Table S3). Most of these gene families corresponded to *B. vulgatus* and *B. uniformis*. As expected, the healthy control group showed characteristic gene families from *P. distasonis*, *R. gnavus* and *Bacteroides* sp., while characteristic gene families of outpatients corresponded to *Bacteroides* and *Eubacterium* in agreement with taxonomic profiles of samples (Table 1, Fig. 2, Supplementary Figure S4).

### Metagenome-assembled genomes (MAGs)

#### MAGs recovery

Assembly-based analysis led to a total 991 bins assembled from healthy control and outpatients recovered from AL metagenomes. These bins were quality-filtered based on completeness and contamination (at least 50% of the genome is represented and contains less than 5% contamination according to previous works^23^. Therefore, a total 381 draft metagenome-assembled genomes (MAGs) of medium to high quality were recovered from healthy controls (n = 129) and outpatients recovered from AL (n = 252) (Supplementary Table S4). Some MAGs were recovered from both healthy controls and outpatients recovered from AL and were classified as “common MAGs”. In contrast, some MAGs were recovered only from one group of samples and were classified as “characteristic MAGs of healthy controls” or “characteristic MAGs of outpatients recovered from AL”. Then, MAGs were classified at taxonomic level. Some MAGs could be identified at strain or species level while other sequences could be correctly identified only at genus or family level (Supplementary Table S3).

The most frequent species recovered from outpatient and healthy control groups were *E. coli* and *P. distasonis*, respectively. As expected, a large number of MAGs belonging to different species of the genus *Prevotella* obtained were recovered from outpatient metagenomes compared to the control group, in agreement with assembly-free taxonomic analysis (Table 1). On the other hand, the majority of MAGs recovered from healthy controls belonged to several genera that are commonly associated with a healthy gut composition such as *Faecalibacterium*, *Faecalibacillus, Akkermansia* or *Copromonas*^24,25^. These results highlight the negative impact that chemotherapy and further colonization by ESBL- and/or carbapenemase-producing *Enterobacterales* exert on the gut microbiota of outpatients, leading to a reduction in microbial diversity.

#### Functional annotation of MAGs

**Figure 4:**
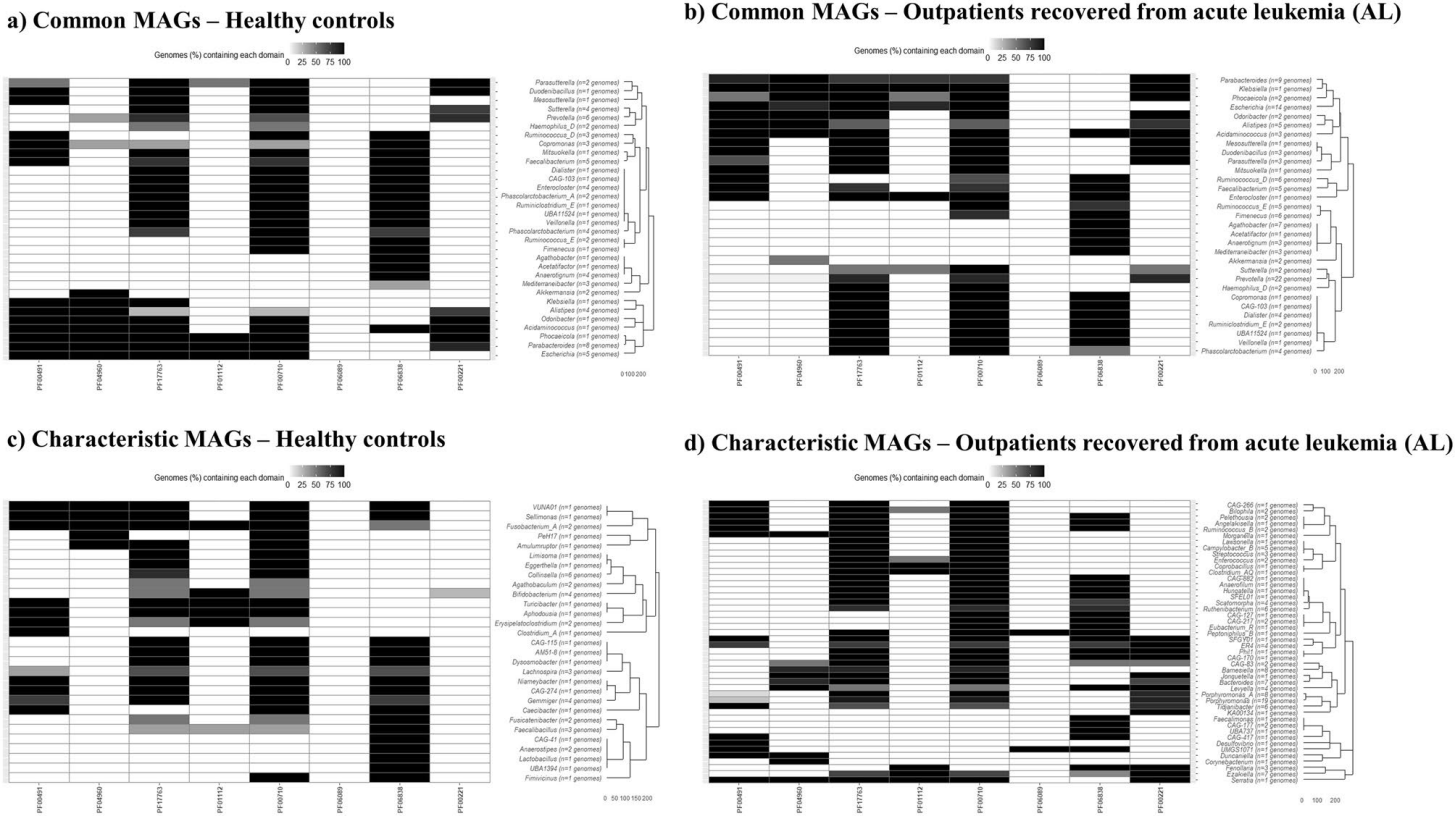
Heatmap showing the presence of different microbial domains involved in the prevention of acute leukemia (indicated as black cells) in metagenome-assembled genomes (MAGs) recovered from the microbiota of outpatients recovered from acute leukemia (AL) or that underwent stem cell transplantation and healthy controls: (**a**) MAGs recovered from healthy controls that were also found in the microbiota of outpatients recovered from AL, (**b**) MAGs from outpatients recovered from AL that were also found in the microbiota of healthy controls, (**c**) MAGs recovered only from healthy controls, (**d**) MAGs recovered only from outpatients. These MAGs were assigned to taxonomic clades (see Supplementary Table S4). **PF00491**: arginase; **PF04960** and **PF17763**: glutaminase; **PF01112**, **PF00710** and **PF06089**: l-asparaginase; **PF06838**: methionine γ-lyase; **PF00221**: phenylalanine ammonia lyase / tyrosine phenol lyase.

These MAGs were also annotated at functional level and the capability to produce therapeutic enzymes was investigated. In this regard, l-asparaginase has been widely used in the treatment of acute lymphoblastic leukemia, and other lymphoid malignancies in combination with other drugs^26,27^. The presence/absence of l-asparaginase and similar enzymes with therapeutic activity was detected in MAGs recovered from healthy controls and outpatients recovered from AL (Fig. 4). These enzymes were annotated in several MAGs of a wide range of genera recovered from both healthy and outpatient metagenomes including *Prevotella*, *Ruminococcus*, *Faecalibacterium*, *Alistipes*, *Akkermansia* (Fig. 4a, b). In general, major differences were observed between both groups of samples. These functional domains showed a greater representation in characteristic MAGs from outpatients than in characteristic MAGs from controls (Fig. 4c, d). These enzymes were detected in at least two MAGs from each genus recovered from the outpatient group (Fig. 4d). Despite differences among groups, most MAGs from a wide range of genera showed glutaminase (**PF17763**) and l-asparaginase (**PF00710**) domains. Interestingly, **PF06089** l-asparaginase domain was detected only in MAGs from *Peptoniphilus B* and novel *Acutalibacteraceae* species *UMGS1071* recovered from outpatients. It is worth noting the absence of the enzyme phenylalanine ammonia lyase / tyrosine phenol lyase (**PF00221**) in all characteristic MAGs recovered from the control group, with the exception *Bifidobacterium* MAGs (Fig. 4c).

As explained, l-asparaginase is an antineoplastic agent that has been widely used in the acute lymphoblastic leukemia chemotherapy^26,27^. Therefore, the presence of l-asparaginase domains in major gut microbial genera may play a positive role in the recovery of outpatients.

### Metabolic interaction network

Finally, to gain a better understanding of metabolic interactions between MAGs recovered from healthy controls and outpatients recovered from AL, potential cross-feeding mechanisms were elucidated in silico according to Belcour et al^19^. In this regard, essential symbionts and alternative symbionts were determined. Essential symbionts comprise key microorganisms that occur in every minimal community of MAGs needed to satisfy one specific metabolic function through metabolic cooperation of these bacteria in colon lumen. In contrast, alternative symbionts comprise those microorganisms that occur only in some of these minimal communities of interacting microbes. Therefore, any of the alternative symbionts can complete the missing metabolic pathways of the minimal microbial community.

Essential symbionts (n = 30) from healthy controls included bifidobacterial like *B. pseudocatenulatum* and *B. bifidum*, *P. distasonis*, *F. prausnitzii* and novel species *Lachnospira* sp000437735 and *Lachnospirales* CAG-274 sp900545305 and AM51-8 sp003478275. Among Alternative symbionts (n = 6) from this group of samples included *R. D bicirculans*, *Gemmiger forcimicilis*, CAG-115 sp00351585, *Prevotella* sp000434975, *Clostridium A leptum* and *Dialister sp003486385*. On the other hand, essential symbionts (n = 28) from outpatients comprised different symbiotic species than those found for controls including novel species *Firmicutes* bacterium CAG-568 sp000434395, *Lachnospiraceae* CAG-882 sp003486385 and *Oscillospiraceae* CAG-170 sp000432135. Alternative symbionts (n = 4) from outpatients included *Parasutterella excrementihominis, Dialister succinatiphilus, Coprobacillus cateniformis and Agathobacter rectalis*. It should be noted that there were no common alternative symbionts between both groups of samples.

**Figure 5:**
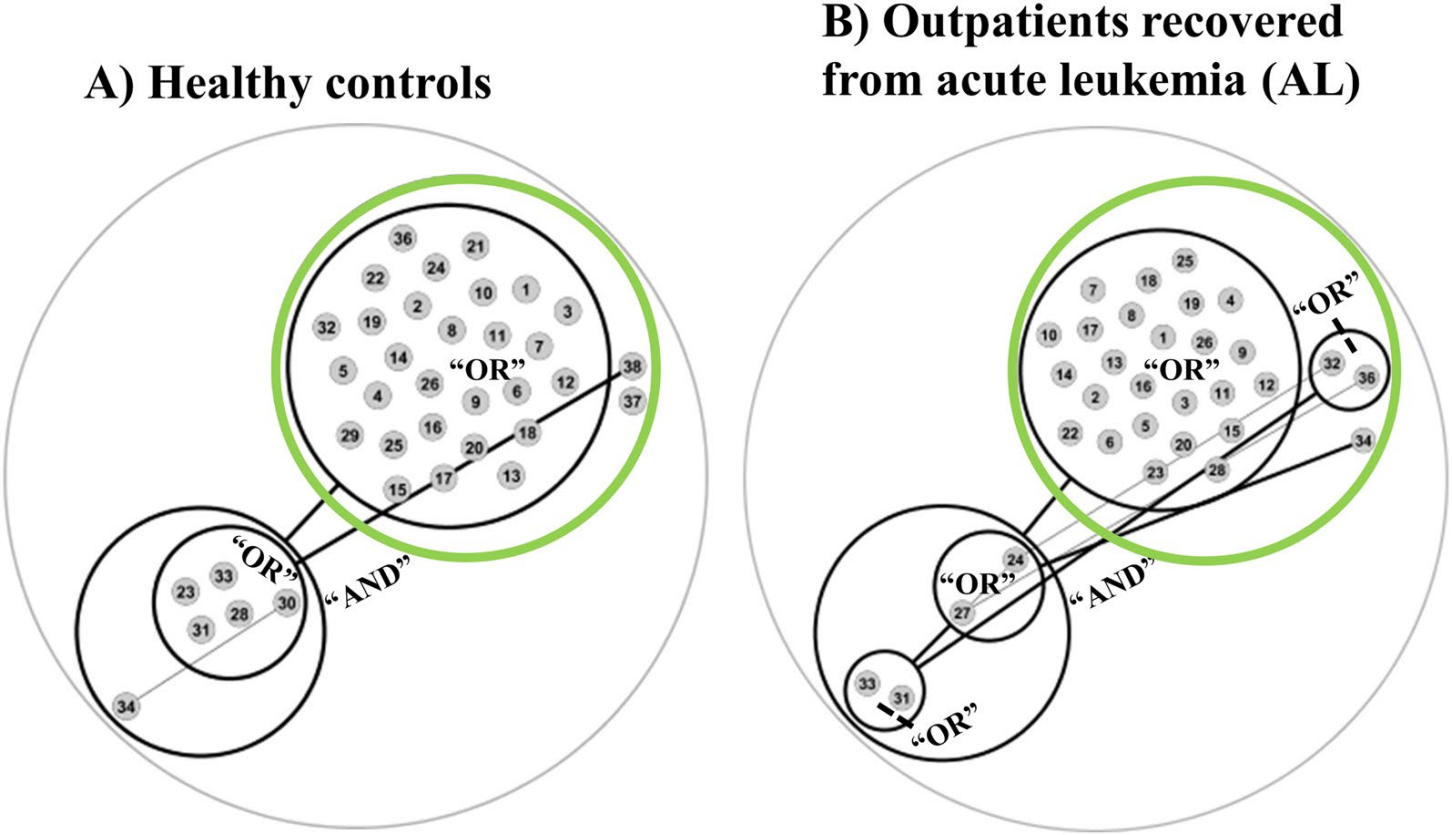
Metabolic network illustrating potential cross-feeding mechanisms between metagenome-assembled genomes (MAGs) recovered from the microbiota of healthy controls (**A**) and outpatients recovered from acute leukemia (AL) (**B**). **MAGs from network **(**A**)** include**: 1: *Bifidobacterium pseudocatenulatum*, 2: *Caecibacter hominis*, 3: *Acidaminococcus provencensis*, 4: *Bifidobacterium bifidum*, 5: *Limosilactobacillus fermentum*, 6: *Sellimonas intestinalis*, 7: *51–20 sp001917175*, 8: *PeH17 sp000435055*, 9: *Lachnospira sp000437735*, 10: *Klebsiella pneumoniae*, 11: *Fusobacterium A mortiferum*, 12: *Parabacteroides distasonis*, 13: *Fusobacterium A mortiferum*, 14: *Enterocloster sp900541315*, 15: *Escherichia coli*, 16: *CAG-274 sp900545305*, 17: *Faecalibacterium prausnitzii G*, 18: *AM51-8 sp003478275*, 19: *UBA1394 sp900538575*, 20: *Scatocola faecipullorum*, 21: *Odoribacter splanchnicus*, 22: *Sutterella wadsworthensis*, 23: *Ruminococcus D bicirculans*, 24: *Phascolarctobacterium faecium*, 25: *Parabacteroides distasonis*, 26: *Akkermansia muciniphila B*, 27: *Mesosutterella multiformis*, 28: *Gemmiger formicilis*, 29: *Alistipes finegoldii*, 30: *CAG-115 sp003531585*, 31: *Prevotella sp000434975*, 32: *Copromonas sp900066535*, 33: *Clostridium A leptum*, 34: *Dialister sp000434475*, 35: *Mitsuokella multacida*, 36: *Aphodousia sp900553105*, 37: *Ruminococcus E bromii B*, 38: *Veillonella parvula A*. **MAGs from network **(**B**) **include**: 1: *Streptococcus anginosus*, 2: *Bifidobacterium piotii*, 3: *Acetatifactor intestinalis*, 4: *Streptococcus oralis V*, 5: *CAG-568 sp000434395*, 6: *CAG-882 sp003486385*, 7: *CAG-267 sp001917135*, 8: *Klebsiella pneumoniae*, 9: *Serratia liquefaciens*, 10: *Ruthenibacterium lactatiformans*, 11: *Escherichia coli*, 12: *Hungatella effluvii*, 13: *Enterocloster clostridioformis*, 14: *Duncaniella*, 15: *Parasutterella*, 16: *CAG-170 sp000432135*, 17: *Bilophila wadsworthia*, 18: *Lawsonella sp018376445*, 19: *SFGY01*, 20: *Jonquetella anthropi*, 21: *Scatomorpha intestinigallinarum*, 22: *Varibaculum*, 23: *Fenollaria sp900539725*, 24: *Parasutterella excrementihominis*, 25: *Phascolarctobacterium faecium*, 26: *Ezakiella coagulans*, 27: *Dialister succinatiphilus*, 28: *ER4 sp000765235*, 29: *UBA11524 sp000437595*, 30: *Pelethousia gallinarum*, 31: *Coprobacillus cateniformis*, 32: *Veillonella parvula A*, 33: *Agathobacter rectalis*, 34: *Levyella massiliensis*, 35: *Ruminococcus D bicirculans*, 36: *Sutterella sp900762445*. Network nodes (i. e. circles containing different communities showing equivalent metabolic functions) are connected by black lines indicating synergistic relationships between communities. Metabolic functions of MAGs from different nodes are needed to achieve the maximum number of end-products from pectin as well as other colonic metabolites (this mutualistic relationship is indicated by the conjunction “AND”). MAGs inside the same node play the same role and could be replaced by other members from the same community (this similar role is indicated by the conjunction “OR”). Essential symbionts are included in the green nodes.

These symbiotic relationships were calculated by determining the number of genes associated to different microbial metabolic activities in MAGs. This parameter was calculated according to Belcour et al^19^, ranging from 194 to 2047 in outpatients recovered from AL, and from 264 to 1991 in healthy controls. Interestingly, the producibility of *myo*-inositol, tartrate and fructoselysine phosphate was observed only in healthy controls. To illustrate these metabolic interactions, a metabolic network of symbiotic bacteria comprising essential and alternative symbionts is provided in Fig. 5.

With regard to the healthy control group, two main microbial communities were observed. The vast majority of bacteria showed equivalent and complementary metabolic functions and potential synergistic relationships with *Ruminococcus E bromii B* and *Veillonella parvula A*. Interestingly, *Ruminococcus bicirculans*, *Gemmiger formicilis*, *Prevotella sp000434975*, *Prevotella sp000434975* and novel *Eubacterium* species CAG-115 sp003531585 comprised one equivalent metabolic community complementary to *Dialister* sp000434475 (Fig. 5A). This community showed potential synergistic interactions with the rest of microbes. On the other hand, similar communities were observed in the outpatient group. In this regard, *Coprobacillus cateniformis* and *Agathobacter rectalis*, and *Parasutterella excrementihominis* and *Dialister succinatiphilus* comprised two equivalent communities that showed complementary metabolic capabilities with the rest of MAGs. In addition, other species like *V. parvula* and *Levyella massiliensis* showed potential interactions with multiple communities (Fig. 5B).

## Discussion

This study shows how the administration of treatments such as intensive chemotherapy affects the microbiota composition in the gut of outpatients with AL. One of the limitations of the study is the disparity in the moments in which the samples were collected. However, 75% of the samples were collected two years after the last chemotherapy cycle was administered, indicating that the abnormalities in the microbiota persist even when the mucosal damage has stopped. These results are consistent with other studies showing a decrease in taxonomic diversity of gut microbiome in the outpatient group^28^, although microbiome composition was more variable in outpatient samples than in healthy controls. Remarkably, a decrease of gut microbiota diversity is generally associated with a high-risk of infectious diseases and was proposed as a marker to predict the potential complications associated with intensive chemotherapy in AL patients^29^.

It should be noted that the samples included in this study correspond to outpatients with AL treated with intensive chemotherapy decolonized from an infection with *Enterobacteriaceae* producing ESBL and/or carbapenemases. Carbapenems, cefepime, and piperacillin/tazobactam are established front-line empiric treatments for febrile neutropenia in patients with hematologic malignancies. In relation to this, other authors have seen that treatment with carbapenems is related to the greatest decrease in *Blautia*^30–32^, as we have observed. Recent studies suggest that the *Lachnospiraceae*, *Ruminococcaceae*, *Bacteroidaceae*, *Prevotellaceae*, and *Clostridiaceae* families, widely distributed in the intestinal tract, were predominant in healthy individuals^33^, but the current study indicates that *Bacteirodaceae*, *Bifidobacteriaceae* and *Lachnospiraceae* families were predominant in our control samples, while the rest of the predominant families were found in lower proportions.

The greatest composition differences were detected from outpatient samples. In this samples, we detected an increase in the relative abundance of *Prevotella* and *Escherichia* to a lesser extent, *Alistipes*. Compared to healthy controls, in the cancer patient’s gut microbiota were reported with increased levels of *Fusobacterium* and *Proteobacteria* (especially *Providencia*), and decreased abundance in the *Clostridiales* order of the *Firmicutes* phylum (*Lachnospiraceae*, *Ruminococcaceae*, and *Faecalibacterium prausnitzii*), and in the *Bacteroidales* order of the *Bacteroidetes* phylum (*Bacteroides*, *Rikenellaceae*)^34^.

In addition, results shown an increase of *Bacteroides vulgatus* in the outpatients gut that increases as treatment time increases. This increase was reported to be caused by treatment with an H2 receptor blocker or proton pump inhibitors and could be related with patients whose treatment regimen included omeprazole or, rarely, pantoprazole^33,35^.

The use of anti-metabolites is common in the treatment of hematological cancers such as leukemias because these neoplastic cells are often auxotrophic to specific amino acids, which makes them susceptibility to such treatments^36^. The anti-metabolites are anticancer drugs with capacity of preventing synthesis or depleting the supply of essential molecules for cancer cell proliferation, such as nitrogenous bases, nucleotides, and amino acids^37^. The therapeutic use of enzymes has been well studied. Amino acid deprivation therapy (AADT) is one such promising strategy characterized by the usage of amino acid depleting enzymes, thus promoting the treatment of cancers. l-asparaginase (l-asparagine amidohydrolase, l-AspL-Asp) was the first microbial enzyme used for AADT and is currently available as a drug highly effective against T cell acute lymphoblastic leukemia (T-ALL). Since then, other enzymes like arginine deiminase, arginase, glutaminase, methionase, lysine oxidase, and phenylalanine ammonia lyase are being explored for cancer treatment^34^. l-Asp has been extensively used and studied because of its relevant potential as an anti-oncological agent and as an acrylamide mitigation agent in the food industry, which is due to its ability to catalyze the hydrolysis of l-asparagine into l-aspartate and ammonia^38,39^.

l-asparaginase (l-Asp) is an important chemotherapeutic agent in the treatment of ALL, but it is not part of the standard chemotherapeutic schemes of AML. However, several in vitro studies have shown that some cell lines and primary AML samples are sensitive to l-Asp to the same extent as ALL cells^40,41^. This sensitivity is partially explained by haploinsufficiency of L asparagine synthetase, which is located in chromosome 7q21.3^42^. More importantly, l-Asp depletes AML cells of glutamine, which is an essential nutrient^43^. In line with these observations, preliminary reports have demonstrated the benefits of l-Asp in monotherapy^44^ or in combination with other drugs^45–47^ in the treatment of relapsed/refractory AML patients. The detection of this enzyme in a high number of MAGs belonging to outpatients who have attained sustained remission allows to hypothesize that these specific changes in the microbiome contributed to the control of the underlying neoplasm and supports the incorporation of l-asp in the therapeutic arsenal of AML.

These enzymes are synthesized by a large number of microorganisms present in the human gut, including patient samples, as demonstrated in this study. Interestingly, novel l-asparaginases with anti-leukemic effect have been discovered via in silico screening of prokariotic genomes and metagenomics^48^, highlighting the potential of next-generation sequencing and bioinformatic methods to elucidate microbial metabolism on leukemia treatment. The present work describes novel applications of metagenome assembly and metabolic modelling to elucidate biological interactions in the microbiota of outpatients recovered from AL, revealing cross-feeding interactions between health-promoting bacteria including *Ruminococcus*, novel *Lachnospiraceae* species and *Veillonella* among others. It has been reported that members of the *Ruminococcaceae* and *Lachnospiraceae* families were still depleted in pediatric survivors of ALL while the abundance of *Ruminococcus* species tended to increase during the chemotherapy regimen in pediatric patients of ALL^17,49^. Metabolic interactions presented in this work constitute a first approach to elucidate microbial metabolism and dynamics in the context of AL. Metabolic synergies between cooperating bacteria could be used to design novel microbiota-oriented interventions to modulate the growth of health promoting microbial communities that may alleviate some of the adverse side effects of chemotherapy.

In conclusion, the present work sheds light on what happens to the microbiota of outpatients once they have finished the antineoplastic treatment in terms of microbial composition, diversity and metabolic interactions. We show that, even when the outpatients were able to be decolonized of MDR bacteria, profound changes were still present in their microbiota. Some of these changes are related to adverse outcomes. For example, the predominance of *Prevotella* species has been associated with acute graft versus host disease, a life-threatening complication following allogeneic stem cell transplant (SCT)^50^. Considering that almost half of the AL patients will experiment a relapse, which is treated with further chemotherapy and SCT, the permanence of these species poses the patients at risk of a GVHD in the event of a relapse. Therefore, the development of strategies for the recovery of the normal microbiota should be a priority in the treatment of patients with AL. The long-term consequences of gut microbiome alterations are unknown. Therefore, further studies including a larger number of patients are required, but the results of the present work may constitute a good starting point for this purpose.

## Material and methods

### Samples collection

A total of 28 samples (21 rectal swabs and 7 faecal samples) from outpatients under follow-up by the Haematology Department of the “Hospital Universitario Central de Asturias” (HUCA) who recovered from AL or high-risk myelodysplastic syndrome treated with intensive chemotherapy, were collected. In this regard, outpatients comprised 19 female and 9 male individuals who were 31 to 72 years old. Outpatients enrolled in this study were diagnosed with ALL (n = 2), AML (n = 25) and high-risk MDS (n = 1). The median time elapsed between the study sample and the last chemotherapy cycle administered was 849 days (interquartile range 564–1290). Metagenomics analysis was performed, for which DNA was extracted from the samples with the QIAamp Fast DNA Stool Mini Kit (Qiagen, Hilden, Germany) and sequenced in a NovaSeq 6000 platform with Illumina technology, to generate 150 bp paired-end reads, as described a previously study by our group (PRJNA914091)^22^;. Besides, outpatient metagenomes were compared to healthy control metagenomes (n = 14) reported by Yachida et al^51^. These control metagenomes (study accession code PRJDB4176) were paired by sex and age and retrieved from the standardised database HumanMetagenomeDB^52^. Control samples were sequenced in a HiSeq2500 platform (Illumina) to generate 150 bp paired-end reads. A table summarising the accession codes of metagenome sequences and demographic characteristics of AL outpatients and healthy controls can be found in Supplementary Material Tables S1 and S2.

### Assembly-free analysis

Raw metagenome sequences were analysed using TORMES v1.3.0^53^, a software tool which implements a comprehensive pipeline for meta-genomics analysis (including quality control of the reads, de novo genome assembly, and screening of antimicrobial resistance encoding genes, among others). Quality filtering was accomplished by Prinseq v0.20.4^54^. Reads with a quality score lower than 25 or with less than 125 bp were excluded from the analysis.

An assembly-free analysis of metagenome sequences was first performed to better characterise low-abundance taxa that might not be assembled. For this purpose, metagenomic clean reads generated by TORMES v1.3.0^53^ were concatenated and analysed following MetaPhlAn 3.0 (v3.0.4) and HUMAnN 3.0 (v3.0.0) pipelines^55,56^. In this regard, ChocoPhlAn (version "mpa_v30_ChocoPhlAn_201901") database containing clade-specific marker genes and UniRef90 (version "uniref90_201901") protein database, were used to perform taxonomic and functional analyses. The abundances of gene families and metabolic pathways were re-normalized and expressed in units of copies per million.

Statistical analyses were performed on R (v.4.2.2). To characterize microbial diversity within a sample and between individuals, alpha and beta diversity estimators were computed using Phyloseq^57^ and Microbiome R packages^58^. Principal Coordinate Analysis (PCoA) and composition barplots were generated using Microbiome R package to illustrate major differences in the microbiota of outpatients and healthy controls. Statistically significant differences (p_adj_ < 0.05) in microbiota composition and microbial gene families and metabolic pathways were calculated using multiple statistical methods designed for microbiome analysis (aldex, ANCOM. ANCOMBC, LEfSe, metagenomeSeq and DESeq2) implemented in microbiomeMarker R package^59–65^. Taxonomic clades and functions classified as significantly differential microbes (microbiome markers) by any of these methods were selected for further analysis.

### Metagenome assembly

A complementary assembly-based analysis of metagenome sequences was carried out to perform a comprehensive characterisation of microbial metabolic activities. The assembly of the filtered reads was performed with MEGAHIT v1.2.9^66^. Maximum k-mer size was set at 127 in order to generate the following series of k-mers: k-21, k-31, k-41, k-51, k-61, k-71, k-81, k-91, k-101, k-111, k-121, k-127). Then, metagenome reads were mapped against the assembly using Bowtie2 v.4.5^67^. Output bam files generated were sorted and indexed. Contigs larger than 1.5 kilobases were binned separately using Metabat2 v.2.2.15^68^. MAGs completeness and contamination was determined using CheckM v.1.1.3 lineage-specific workflow^69^. In this regard, MAGs showing completeness lower than 50% and contamination higher than 5% were discarded according to previous works^18,70^. Taxonomic classification of MAGs was performed using GTDB-Tk v2.1.1 software^71^. Open reading frames (ORFs) of MAGs were determined using Prodigal v.2.6.3 and annotated using HMMER software and Pfam database^72^.

Further statistical analyses were performed on R (v.4.2.2). Hierarchical clustering of MAGs was carried out considering the distribution of Pfam domains corresponding to enzymes that are positively associated to leukemia remission according to previous studies^26,27^: **PF00491** arginase; **PF04960** and **PF17763** glutaminases; **PF01112**, **PF00710** and **PF06089** l-asparaginases; **PF06838** methionine γ-lyase; and **PF00221** phenylalanine ammonia lyase / tyrosine phenol lyase. Clusters illustrating the presence and absence of these Pfam domains in MAGs were calculated by the complete linkage method using the basic R function “hclust”.

### Metabolic modelling

The last step of the bioinformatic analysis involved metabolic modelling of MAGs recovered from AL outpatients and healthy controls to elucidate potential metabolic interactions between microbial communities. MAGs were first annotated using Prokka v1.14.6 and standard Genbank files (.gbk) files containing sequences and annotations were used as input for metage2metabo v1.5.0 software. A “seeds” file containing different nutrients that may be present in human gut according to previous studies was provided to perform the simulations in given nutritional conditions^18,19^. Different simulations were performed for AL outpatients and healthy controls.

### Ethics declarations

The present study was reviewed and approved by Comité de Ética de la Investigación del Principado de Asturias (CEImPA) with number 204/17) Written informed consent to participate in this study was provided by the participants.

### Supplementary Information

Supplementary Information.

## Data Availability

The datasets presented in this study can be found in Sequence Read Archive (SRA) of NCBI (https://www.ncbi.nlm.nih.goc/sra). Under BioProject code PRJNA914091, with BioSample codes from SAMN32318268 to SAMN32318297.

## References

[1] Sender R, Fuchs S, Milo R (2016). Revised estimates for the number of human and bacteria cells in the body. PLoS Biol..

[2] Sommer F, Bäckhed F (2013). The gut microbiota–masters of host development and physiology. Nat Rev Microbiol..

[3] Ley RE, Hamady M, Lozupone C, Turnbaugh PJ, Ramey RR, Bircher JS (2008). Evolution of mammals and their gut microbes. Science.

[4] Dzutsev A, Goldszmid RS, Viaud S, Zitvogel L, Trinchieri G (2015). The role of the microbiota in inflammation, carcinogenesis, and cancer therapy. Eur. J. Immunol..

[5] Speck NA, Gilliland DG (2002). Core-binding factors in haematopoiesis and leukaemia. Nat Rev Cancer..

[6] Döhner H, Weisdorf DJ, Bloomfield CD (2015). Acute myeloid leukemia. N. Engl. J. Med..

[7] Inaba H, Greaves M, Mullighan CG (2013). Acute lymphoblastic leukaemia. Lancet Lond. Engl..

[8] Sung H, Ferlay J, Siegel RL, Laversanne M, Soerjomataram I, Jemal A (2021). Global cancer statistics 2020: GLOBOCAN estimates of incidence and mortality worldwide for 36 cancers in 185 countries. CA Cancer J. Clin..

[9] Jewani, K., Boddu, K., Gumani, P., Solapure, K. Detection of diseases via blood analysis using Image processing Techniques. *Proc. 2018 International Conference on Smart City and Emerging Technology (ICSCET)* [Internet]. 2018 [citado 24 de octubre de 2023]. 1–4. Disponible en: https://ieeexplore.ieee.org/document/8537364

[10] Bernard E, Tuechler H, Greenberg PL, Hasserjian RP, Arango Ossa JE, Nannya Y (2022). Molecular international prognostic scoring system for myelodysplastic syndromes. NEJM Evid..

[11] Zhang H, Zhan Q, Wang X, Gao F, Yu J, Wang J (2022). TLS/FUS-ERG fusion gene in acute leukemia and myelodysplastic syndrome evolved to acute leukemia: Report of six cases and a literature review. Ann. Hematol..

[12] Burger JA, O’Brien S (2018). Evolution of CLL treatment-from chemoimmunotherapy to targeted and individualized therapy. Nat. Rev. Clin Oncol..

[13] Terwilliger T, Abdul-Hay M (2017). Acute lymphoblastic leukemia: A comprehensive review and 2017 update. Blood Cancer J..

[14] Stein EM, Tallman MS (2016). Emerging therapeutic drugs for AML. Blood.

[15] Jabbour E, Kantarjian H (2020). Chronic myeloid leukemia: 2020 update on diagnosis, therapy and monitoring. Am. J. Hematol..

[16] Ciftciler R, Ciftciler AE (2022). The importance of microbiota in hematology. Transfus Apher Sci. Off. J. World Apher. Assoc. Off. J. Eur. Soc. Haemapheresis.

[17] Rajagopala SV, Singh H, Yu Y, Zabokrtsky KB, Torralba MG, Moncera KJ (2020). Persistent gut microbial dysbiosis in children with acute lymphoblastic leukemia (ALL) during chemotherapy. Microb. Ecol..

[18] Sabater C, Calvete-Torre I, Ruiz L, Margolles A (2022). Arabinoxylan and pectin metabolism in Crohn’s Disease microbiota: An in silico study. Int. J. Mol. Sci..

[19] Belcour A, Frioux C, Aite M, Bretaudeau A, Hildebrand F, Siegel A (2020). Metage2Metabo, microbiota-scale metabolic complementarity for the identification of key species. eLife..

[20] Gyarmati P, Kjellander C, Aust C, Song Y, Öhrmalm L, Giske CG (2016). Metagenomic analysis of bloodstream infections in patients with acute leukemia and therapy-induced neutropenia. Sci. Rep..

[21] Rechenberger J, Samaras P, Jarzab A, Behr J, Frejno M, Djukovic A (2019). Challenges in clinical metaproteomics highlighted by the analysis of acute leukemia patients with gut colonization by multidrug-resistant enterobacteriaceae. Proteomes.

[22] Lumbreras-Iglesias P, Sabater C, Moreno FA, de Ugarriza LP, Fernández-Verdugo A, Margolles A (2023). Evaluation of a shotgun metagenomics approach for detection of ESBL- and/or carbapenemase-producing enterobacterales in culture negative patients recovered from acute leukemia. Microorganisms.

[23] Bowers RM, Kyrpides NC, Stepanauskas R, Harmon-Smith M, Doud D, Reddy TBK (2017). Minimum information about a single amplified genome (MISAG) and a metagenome-assembled genome (MIMAG) of bacteria and archaea. Nat. Biotechnol..

[24] Sabater C, Calvete-Torre I, Villamiel M, Moreno FJ, Margolles A, Ruiz L (2021). Vegetable waste and by-products to feed a healthy gut microbiota: Current evidence, machine learning and computational tools to design novel microbiome-targeted foods. Trends Food Sci. Technol..

[25] Li Y, Yang H, Xu L, Wang Z, Zhao Y, Chen X (2018). Effects of dietary fiber levels on cecal microbiota composition in geese. Asian-Australas J. Anim. Sci..

[26] Husain I, Sharma A, Kumar S, Malik F (2016). Purification and characterization of glutaminase free asparaginase from *Pseudomonas otitidis*: Induce apoptosis in human leukemia MOLT-4 cells. Biochimie.

[27] Vala AK, Sachaniya B, Dudhagara D, Panseriya HZ, Gosai H, Rawal R (2018). Characterization of L-asparaginase from marine-derived *Aspergillus niger* AKV-MKBU, its antiproliferative activity and bench scale production using industrial waste. Int. J. Biol. Macromol..

[28] Fattizzo B, Cavallaro F, Folino F, Barcellini W (2021). Recent insights into the role of the microbiome in malignant and benign hematologic diseases. Crit. Rev. Oncol. Hematol..

[29] Rattanathammethee T, Tuitemwong P, Thiennimitr P, Sarichai P, Na Pombejra S, Piriyakhuntorn P (2020). Gut microbiota profiles of treatment-naïve adult acute myeloid leukemia patients with neutropenic fever during intensive chemotherapy. PLoS ONE.

[30] Bai L, Zhou P, Li D, Ju X (2017). Changes in the gastrointestinal microbiota of children with acute lymphoblastic leukaemia and its association with antibiotics in the short term. J. Med. Microbiol..

[31] Han L, Zhang H, Chen S, Zhou L, Li Y, Zhao K (2019). Intestinal microbiota can predict acute graft-versus-host disease following allogeneic hematopoietic stem cell transplantation. Biol. Blood Marrow Transpl. J. Am. Soc. Blood Marrow Transpl..

[32] Ford CD, Coombs J, Stofer MG, Lopansri BK, Webb BJ, Ostronoff F (2019). Decrease in vancomycin-resistant Enterococcus colonization associated with a reduction in carbapenem use as empiric therapy for febrile neutropenia in patients with acute leukemia. Infect. Control Hosp. Epidemiol. julio de.

[33] Akhremchuk KV, Skapavets KY, Akhremchuk AE, Kirsanava NP, Sidarenka AV, Valentovich LN (2022). Gut microbiome of healthy people and patients with hematological malignancies in Belarus. Microbiol. Indep. Res. J. MIR J..

[34] Dhankhar R, Gupta V, Kumar S, Kapoor RK, Gulati P (2020). Microbial enzymes for deprivation of amino acid metabolism in malignant cells: biological strategy for cancer treatment. Appl. Microbiol. Biotechnol..

[35] Serebrova S, Dobrovolskiy O (2007). Peptic ulcer therapy and problems of microecology of the gastrointestinal tract. Russ. Med. J..

[36] Fung MKL, Chan GCF (2017). Drug-induced amino acid deprivation as strategy for cancer therapy. J. Hematol. Oncol. J. Hematol. Oncol..

[37] Vander Heiden MG (2011). Targeting cancer metabolism: A therapeutic window opens. Nat. Rev. Drug Discov..

[38] Sharma D, Singh K, Singh K, Mishra A (2019). Insights into the microbial L-asparaginases: From production to practical applications. Curr. Protein Pept. Sci..

[39] Chand S, Mahajan RV, Prasad JP, Sahoo DK, Mihooliya KN, Dhar MS (2020). A comprehensive review on microbial l-asparaginase: Bioprocessing, characterization, and industrial applications. Biotechnol. Appl. Biochem..

[40] Jun SA, Sepiashvili L, Kislinger T, Minden MD (2011). Investigating the potential use of L-asparaginase in myeloid leukemia. Blood.

[41] Okada S, Hongo T, Yamada S, Watanabe C, Fujii Y, Ohzeki T (2003). In vitro efficacy of L-asparaginase in childhood acute myeloid leukaemia. Br. J. Haematol..

[42] Bertuccio SN, Serravalle S, Astolfi A, Lonetti A, Indio V, Leszl A (2017). Identification of a cytogenetic and molecular subgroup of acute myeloid leukemias showing sensitivity to L-asparaginase. Oncotarget.

[43] Willems L, Jacque N, Jacquel A, Neveux N, Maciel TT, Lambert M (2013). Inhibiting glutamine uptake represents an attractive new strategy for treating acute myeloid leukemia. Blood.

[44] Emadi A, Law JY, Strovel ET, Lapidus RG, Jeng LJB, Lee M (2018). Asparaginase *Erwinia chrysanthemi* effectively depletes plasma glutamine in adult patients with relapsed/refractory acute myeloid leukemia. Cancer Chemother. Pharmacol..

[45] Buaboonnam J, Cao X, Pauley JL, Pui CH, Ribeiro RC, Rubnitz JE (2013). Sequential administration of methotrexate and asparaginase in relapsed or refractory pediatric acute myeloid leukemia. Pediatr. Blood Cancer.

[46] Capizzi RL, Davis R, Powell B, Cuttner J, Ellison RR, Cooper MR (1988). Synergy between high-dose cytarabine and asparaginase in the treatment of adults with refractory and relapsed acute myelogenous leukemia—a cancer and leukemia group b study. J. Clin. Oncol. Off. J. Am. Soc. Clin. Oncol..

[47] Wells RJ, Woods WG, Lampkin BC, Nesbit ME, Lee JW, Buckley JD (1993). Impact of high-dose cytarabine and asparaginase intensification on childhood acute myeloid leukemia: A report from the childrens cancer group. J. Clin. Oncol. Off. J. Am. Soc. Clin. Oncol..

[48] Sobat M, Asad S, Kabiri M, Mehrshad M (2021). Metagenomic discovery and functional validation of L-asparaginases with anti-leukemic effect from the Caspian Sea. iScience..

[49] Thomas R, Wong WSW, Saadon R, Vilboux T, Deeken J, Niederhuber J (2020). Gut microbial composition difference between pediatric ALL survivors and siblings. Pediatr. Hematol. Oncol..

[50] Zargari Marandi R, Jørgensen M, Ilett EE, Nørgaard JC, Noguera-Julian M, Paredes R (2022). Pre-transplant prediction of acute graft-versus-host disease using the gut microbiome. Cells..

[51] Yachida S, Mizutani S, Shiroma H, Shiba S, Nakajima T, Sakamoto T (2019). Metagenomic and metabolomic analyses reveal distinct stage-specific phenotypes of the gut microbiota in colorectal cancer. Nat Med..

[52] Kasmanas JC, Bartholomäus A, Corrêa FB, Tal T, Jehmlich N, Herberth G (2020). HumanMetagenomeDB: A public repository of curated and standardized metadata for human metagenomes. Nucl. Acids Res..

[53] Quijada NM, Rodríguez-Lázaro D, Eiros JM, Hernández M (2019). TORMES: An automated pipeline for whole bacterial genome analysis. Bioinf. Oxf. Engl..

[54] Schmieder R, Edwards R (2011). Quality control and preprocessing of metagenomic datasets. Bioinf. Oxf. Engl..

[55] Franzosa EA, McIver LJ, Rahnavard G, Thompson LR, Schirmer M, Weingart G (2018). Species-level functional profiling of metagenomes and metatranscriptomes. Nat. Methods.

[56] Truong DT, Tett A, Pasolli E, Huttenhower C, Segata N (2017). Microbial strain-level population structure and genetic diversity from metagenomes. Genome Res..

[57] McMurdie PJ, Holmes S (2013). phyloseq: An R package for reproducible interactive analysis and graphics of microbiome census data. PloS One..

[58] Lahti, L., & Shetty, S. Tools for microbiome analysis in R. version 2.1.24 [Internet]. 2017. Disponible en: https://microbiome.github.io/tutorials/

[59] Cao Y, Dong Q, Wang D, Zhang P, Liu Y, Niu C (2022). microbiomeMarker: An R/Bioconductor package for microbiome marker identification and visualization. Bioinf. Oxf Engl..

[60] Fernandes AD, Reid JN, Macklaim JM, McMurrough TA, Edgell DR, Gloor GB (2014). Unifying the analysis of high-throughput sequencing datasets: characterizing RNA-seq, 16S rRNA gene sequencing and selective growth experiments by compositional data analysis. Microbiome.

[61] Lin H, Peddada SD (2020). Analysis of compositions of microbiomes with bias correction. Nat. Commun..

[62] Love MI, Huber W, Anders S (2014). Moderated estimation of fold change and dispersion for RNA-seq data with DESeq2. Genome Biol..

[63] Mandal S, Van Treuren W, White RA, Eggesbø M, Knight R, Peddada SD (2015). Analysis of composition of microbiomes: A novel method for studying microbial composition. Microb. Ecol. Health Dis..

[64] Paulson JN, Stine OC, Bravo HC, Pop M (2013). Differential abundance analysis for microbial marker-gene surveys. Nat Methods..

[65] Segata N, Izard J, Waldron L, Gevers D, Miropolsky L, Garrett WS (2011). Metagenomic biomarker discovery and explanation. Genome Biol..

[66] Li D, Liu CM, Luo R, Sadakane K, Lam TW (2015). MEGAHIT: An ultra-fast single-node solution for large and complex metagenomics assembly via succinct de Bruijn graph. Bioinf. Oxf. Engl..

[67] Langmead B, Wilks C, Antonescu V, Charles R (2019). Scaling read aligners to hundreds of threads on general-purpose processors. Bioinformatics.

[68] Kang DD, Li F, Kirton E, Thomas A, Egan R, An H (2019). MetaBAT 2: An adaptive binning algorithm for robust and efficient genome reconstruction from metagenome assemblies. PeerJ.

[69] Parks DH, Imelfort M, Skennerton CT, Hugenholtz P, Tyson GW (2015). CheckM: Assessing the quality of microbial genomes recovered from isolates, single cells, and metagenomes. Genome Res..

[70] Asnicar F, Berry SE, Valdes AM, Nguyen LH, Piccinno G, Drew DA (2021). Microbiome connections with host metabolism and habitual diet from 1098 deeply phenotyped individuals. Nat Med..

[71] Chaumeil PA, Mussig AJ, Hugenholtz P, Parks DH (2019). GTDB-Tk: A toolkit to classify genomes with the genome taxonomy database. Bioinf. Oxf. Engl..

[72] Mistry J, Chuguransky S, Williams L, Qureshi M, Salazar GA, Sonnhammer ELL (2021). Pfam: The protein families database in 2021. Nucl. Acids Res..

