# Author Correction: Study of the intestinal microbiota composition and the effect of treatment with intensive chemotherapy in patients recovered from acute leukemia

**DOI:** 10.1038/s41598-024-58097-5

**Published:** 2024-04-03

**Authors:** Xenia Vázquez, Pilar Lumbreras-Iglesias, M. Rosario Rodicio, Javier Fernández, Teresa Bernal, Ainhoa Fernández Moreno, Paula López de Ugarriza, Ana Fernández-Verdugo, Abelardo Margolles, Carlos Sabater

**Affiliations:** 1 Traslational Microbiology Group, Instituto de Investigación Sanitaria del Principado de Asturias (ISPA), Oviedo, Spain; 2 Dairy Research Institute of Asturias (IPLA), Spanish National Research Council, (CSIC), Villaviciosa, Asturias Spain; 3 Department of Clinical Microbiology, Hospital Universitario Central de Asturias (HUCA), Oviedo, Spain; 4 Department of Functional Biology, Microbiology Area, University of Oviedo, Oviedo, Spain; 5 Research & Innovation, Artificial Intelligence and Statistical Department, Pragmatech AI Solutions, Oviedo, Spain; 6 Centro de Investigación Biomédica en Red-Enfermedades Respiratorias, Madrid, Spain; 7 Department of Hematology Instituto de Investigación Sanitaria del Principado de Asturias (ISPA), Instituto de Oncología del Principado de Asturias (IUOPA), Hospital Universitario Central de Asturias (HUCA), 33011 Oviedo, Spain; 8 Instituto de Investigación Sanitaria del Principado de Asturias (ISPA), MicroHealth Group, Oviedo, Spain

Correction to: *Scientific Reports* 10.1038/s41598-024-56054-w, published online 07 March 2024

The original version of this Article contained an error in the Acknowledgements section.

“This research was funded by Pfizer as an Independent Scientific Research Grant; by project FIS PI21/01590 (Fondo de Investigación Sanitaria, Instituto de Salud Carlos III, Ministerio de Economía y Competitividad, Spain), co-funded by the European Regional Development Fund of the European Union: A Way to Making Europe; P.L. was founded by the Regional Ministry of Science of Asturias (GRUPIN IDI/2022/000033).”

now reads

“This study has been funded by Instituto de Salud Carlos III (ISCIII) through the project “FIS PI21/01590” and co-funded by the European Union. This research was funded by Pfizer as an Independent Scientific Research Grant. P.L. was founded by the Regional Ministry of Science of Asturias (GRUPIN IDI/2022/000033).”

The original Article has been corrected.

