# Supplementary material for: Study of the intestinal microbiota composition and the effect of treatment with intensive chemotherapy in patients recovered from acute leukemia
Source: Sci Rep. 2024 Mar 7;14:5585. doi: 10.1038/s41598-024-56054-w (PMC10920697; doi:10.1038/s41598-024-56054-w)
Supplement: Supplementary file 1 — Supplementary Information. [file 41598_2024_56054_MOESM1_ESM.docx]

Supplementary Material

**Study of the intestinal microbiota composition and the effect of treatment with intensive chemotherapy in patients recovered from acute leukemia**

Xenia Vázquez^1,2^, Pilar Lumbreras-Iglesias^1,3,7^, M. Rosario Rodicio ^1,4^, Javier Fernández^1,3,5,6^, Teresa Bernal^7^, Ainhoa Fernández Moreno^7^, Paula López de Ugarriza^7^, Ana Fernández-Verdugo^1,3^, Abelardo Margolles^2,8^, Carlos Sabater^2,8,*^

*** Correspondence:** Corresponding Author: carlos.sabater@ipla.csic.es

# Supplementary Data

# Supplementary Figures and Tables

# Supplementary Table S1. Accession codes and demographic characteristics of outpatients recovered from acute leukemia (AL) and who had been colonized by ESBL- and/or carbapenemase-producing *Enterobacterales*. ESBL: extended spectrum beta-lactamase; AML: acute myeloid leukemia; MDS: myelodysplastic syndrome; ALL: acute lymphoblastic leukemia; CR: complete remission.

| **Subject_ID** | **Study accession** | **Sample Accession** | **Experiment accession** | **Run accession** | **Sample type** | **Age** | **Sex** | **Underlying Disease/Status/Time from Administration of the Last Cycle of Chemotherapy (Days)** | **Sample** |
| --- | --- | --- | --- | --- | --- | --- | --- | --- | --- |
| SMet_1 | PRJNA914091 | SAMN32318268 | SRX18811972 | SRR22853208 | Outpatient | 53 | Male | AML/CR/1293 | Feces |
| SMet_2 | PRJNA914091 | SAMN32318269 | SRX18811973 | SRR22853207 | Outpatient | 57 | Female | AML/CR/537 | Rectal swab |
| SMet_3 | PRJNA914091 | SAMN32318270 | SRX18811984 | SRR22853196 | Outpatient | 59 | Male | AML/CR/1215 | Rectal swab |
| SMet_4 | PRJNA914091 | SAMN32318271 | SRX18811995 | SRR22853185 | Outpatient | 69 | Female | AML/CR/795 | Rectal swab |
| SMet_6 | PRJNA914091 | SAMN32318273 | SRX18811997 | SRR22853183 | Outpatient | 47 | Female | AML/CR/477 | Rectal swab |
| SMet_7 | PRJNA914091 | SAMN32318274 | SRX18811998 | SRR22853182 | Outpatient | 74 | Female | AML/CR/834 | Rectal swab |
| SMet_8 | PRJNA914091 | SAMN32318275 | SRX18811999 | SRR22853181 | Outpatient | 47 | Female | AML/CR/588 | Rectal swab |
| SMet_9 | PRJNA914091 | SAMN32318276 | SRX18811999 | SRR22853180 | Outpatient | 31 | Male | AML/CR/1359 | Rectal swab |
| SMet_10 | PRJNA914091 | SAMN32318277 | SRX18812001 | SRR22853179 | Outpatient | 46 | Female | AML/CR/1377 | Rectal swab |
| SMet_11 | PRJNA914091 | SAMN32318278 | SRX18811974 | SRR22853206 | Outpatient | 70 | Male | AML/CR/1263 | Rectal swab |
| SMet_12 | PRJNA914091 | SAMN32318279 | SRX18811975 | SRR22853205 | Outpatient | 39 | Female | AML/CR/867 | Rectal swab |
| SMet_13 | PRJNA914091 | SAMN32318280 | SRX18811976 | SRR22853204 | Outpatient | 56 | Female | AML/CR/729 | Rectal swab |
| SMet_14 | PRJNA914091 | SAMN32318281 | SRX18811977 | SRR22853203 | Outpatient | 50 | Female | AML/CR/351 | Rectal swab |
| SMet_15 | PRJNA914091 | SAMN32318282 | SRX18811978 | SRR22853202 | Outpatient | 49 | Female | AML/CR/1098 | Rectal swab |
| SMet_16 | PRJNA914091 | SAMN32318283 | SRX18811979 | SRR22853201 | Outpatient | 43 | Female | AML/CR/405 | Rectal swab |
| SMet_17 | PRJNA914091 | SAMN32318284 | SRX18811979 | SRR22853200 | Outpatient | 68 | Female | AML/CR/1419 | Rectal swab |
| SMet_18 | PRJNA914091 | SAMN32318285 | SRX18811981 | SRR22853199 | Outpatient | 51 | Male | MDS/CR/630 | Rectal swab |
| SMet_19 | PRJNA914091 | SAMN32318286 | SRX18811982 | SRR22853198 | Outpatient | 61 | Female | AML/CR/831 | Rectal swab |
| SMet_20 | PRJNA914091 | SAMN32318287 | SRX18811983 | SRR22853197 | Outpatient | 58 | Male | ALL/CR/1218 | Rectal swab |
| SMet_21 | PRJNA914091 | SAMN32318288 | SRX18811985 | SRR22853195 | Outpatient | 39 | Female | ALL/CR/51 | Rectal swab |
| SMet_22 | PRJNA914091 | SAMN32318289 | SRX18811986 | SRR22853194 | Outpatient | 67 | Male | AML/CR/864 | Rectal swab |
| SMet_23 | PRJNA914091 | SAMN32318290 | SRX18811987 | SRR22853193 | Outpatient | 72 | Female | AML/CR/303 | Rectal swab |
| SMet_24 | PRJNA914091 | SAMN32318291 | SRX18811988 | SRR22853192 | Outpatient | 54 | Male | AML/CR/1398 | Feces |
| SMet_25 | PRJNA914091 | SAMN32318292 | SRX18811989 | SRR22853191 | Outpatient | 65 | Female | AML/CR/588 | Feces |
| SMet_26 | PRJNA914091 | SAMN32318293 | SRX18811990 | SRR22853190 | Outpatient | 57 | Female | AML/CR/1314 | Feces |
| SMet_28 | PRJNA914091 | SAMN32318295 | SRX18811992 | SRR22853188 | Outpatient | 60 | Female | AML/CR/1428 | Feces |
| SMet_29 | PRJNA914091 | SAMN32318296 | SRX18811993 | SRR22853187 | Outpatient | 69 | Female | AML/CR/1341 | Feces |
| SMet_30 | PRJNA914091 | SAMN32318297 | SRX18811994 | SRR22853186 | Outpatient | 48 | Male | AML/CR/411 | Feces |

**Supplementary Table S2**. Accession codes and demographic characteristics of healthy controls.

| **Subject_ID** | **Study accession** | **Sample Accession** | **Experiment accession** | **Run accession** | **Sample type** | **Age** | **Sex** |
| --- | --- | --- | --- | --- | --- | --- | --- |
| 11524 | PRJDB4176 | SAMD00164950 | DRX162252 | DRR171640 | Healthy control | 31 | Male |
| 12396 | PRJDB4176 | SAMD00165033 | DRX162335 | DRR171723 | Healthy control | 38 | Female |
| 12197 | PRJDB4176 | SAMD00165025 | DRX162327 | DRR171715 | Healthy control | 43 | Male |
| 10465 | PRJDB4176 | SAMD00114809 | DRX162405 | DRR171793 | Healthy control | 47 | Female |
| 11062 | PRJDB4176 | SAMD00164856 | DRX162158 | DRR171546 | Healthy control | 48 | Male |
| 10353 | PRJDB4176 | SAMD00114783 | DRX120281 | DRR127537 | Healthy control | 50 | Female |
| 10510 | PRJDB4176 | SAMD00114824 | DRX162419 | DRR171807 | Healthy control | 52 | Male |
| 11638 | PRJDB4176 | SAMD00164957 | DRX162259 | DRR171647 | Healthy control | 57 | Female |
| 11927 | PRJDB4176 | SAMD00164996 | DRX162298 | DRR171686 | Healthy control | 58 | Male |
| 10354 | PRJDB4176 | SAMD00114784 | DRX162384 | DRR171772 | Healthy control | 60 | Female |
| 10486 | PRJDB4176 | SAMD00114829 | DRX120327 | DRR127583 | Healthy control | 65 | Female |
| 10508 | PRJDB4176 | [SAMD00114858](https://www.ebi.ac.uk/ena/browser/view/SAMD00114858) | [DRX162445](https://www.ebi.ac.uk/ena/browser/view/DRX162445) | [DRR171833](https://www.ebi.ac.uk/ena/browser/view/DRR171833) | Healthy control | 67 | Male |
| 10273 | PRJDB4176 | [SAMD00114991](https://www.ebi.ac.uk/ena/browser/view/SAMD00114991) | [DRX162547](https://www.ebi.ac.uk/ena/browser/view/DRX162547) | [DRR171935](https://www.ebi.ac.uk/ena/browser/view/DRR171935) | Healthy control | 69 | Female |
| 10221 | PRJDB4176 | [SAMD00114976](https://www.ebi.ac.uk/ena/browser/view/SAMD00114976) | [DRX162535](https://www.ebi.ac.uk/ena/browser/view/DRX162535) | [DRR171923](https://www.ebi.ac.uk/ena/browser/view/DRR171923) | Healthy control | 70 | Male |

**Supplementary Table S3.** Number of microbial gene families and metabolic pathways showing the highest abundances in outpatients recovered from acute leukemia (AL) and healthy controls. These gene families are summarised by bacterial species.

| **Microbial genes higher in healthy controls** | | **Microbial genes higher in outpatients recovered from acute leukemia (AL)** | |
| --- | --- | --- | --- |
| **Taxa** | **Frequency** | **Taxa** | **Frequency** |
| *Bacteroides vulgatus* | 1497 | *Bacteroides uniformis* | 50 |
| *Bacteroides uniformis* | 45 | *Bacteroides ovatus* | 3 |
| *Parabacteroides distasonis* | 10 | *Eubacterium rectale* | 3 |
| *Bacteroides thetaiotaomicron* | 5 | *Bacteroides vulgatus* | 2 |
| *Ruminococcus gnavus* | 5 | **Total** | **58** |
| *Bacteroides ovatus* | 3 |  |  |
| **Total** | **1565** |  |  |

**Supplementary Table S4.** Number of metagenome-assembled genomes (MAGs, n=381) recovered from the microbiota of outpatients recovered from acute leukemia (AL) and healthy controls. MAGs were identified at different taxonomic levels.

| **MAGs (n=381) recovered from** | | | |
| --- | --- | --- | --- |
| **Healthy controls** | | **Outpatients recovered from acute leukemia (AL)** | |
| **Taxa** | **Frequency** | **Taxa** | **Frequency** |
| *Parabacteroides distasonis* | 8 | *Escherichia coli* | 14 |
| *Escherichia coli* | 5 | *Barnesiella intestinihominis* | 8 |
| *Faecalibacterium prausnitzii G* | 5 | *Porphyromonas* | 8 |
| *Anaerotignum faecicola* | 4 | *Agathobacter rectalis* | 7 |
| *Alistipes putredinis* | 3 | *Parabacteroides distasonis* | 7 |
| *Collinsella* | 3 | *Fimenecus sp000432435* | 6 |
| *Copromonas sp900066535* | 3 | *Ruminococcus D bicirculans* | 6 |
| *Faecalibacillus intestinalis* | 3 | *Ruthenibacterium lactatiformans* | 6 |
| *Mediterraneibacter torques* | 3 | *Tidjanibacter inops A* | 6 |
| *Ruminococcus D bicirculans* | 3 | *Prevotella bivia* | 5 |
| *Agathobaculum butyriciproducens* | 2 | *Ruminococcus E bromii B* | 5 |
| *Anaerostipes hadrus* | 2 | *ER4 sp000765235* | 4 |
| *Bifidobacterium pseudocatenulatum* | 2 | *Ezakiella coagulans* | 4 |
| *Enterocloster sp001517625* | 2 | *Levyella massiliensis* | 4 |
| *Enterocloster sp900541315* | 2 | *Phascolarctobacterium faecium* | 4 |
| *Erysipelatoclostridium ramosum* | 2 | *Acidaminococcus intestini* | 3 |
| *Fusicatenibacter saccharivorans* | 2 | *Bacteroides uniformis* | 3 |
| *Fusobacterium A mortiferum* | 2 | *Campylobacter B hominis* | 3 |
| *Gemmiger formicilis* | 2 | *Ezakiella sp900540185* | 3 |
| *Gemmiger qucibialis* | 2 | *Faecalibacterium prausnitzii G* | 3 |
| *Phascolarctobacterium faecium* | 2 | *Porphyromonas A somerae* | 3 |
| *Phascolarctobacterium A succinatutens* | 2 | *Porphyromonas A sp001808555* | 3 |
| *Prevotella sp000434975* | 2 | *Prevotella colorans* | 3 |
| *Ruminococcus E bromii B* | 2 | *Prevotella disiens* | 3 |
| *Sutterella wadsworthensis A* | 2 | *Scatomorpha intestinigallinarum* | 3 |
| *51-20 sp001917175* | 1 | *51-20 sp001917175* | 2 |
| *Acetatifactor sp900066565* | 1 | *Alistipes finegoldii* | 2 |
| *Acidaminococcus provencensis* | 1 | *Anaerotignum sp001304995* | 2 |
| *Agathobacter rectalis* | 1 | *Bacteroides thetaiotaomicron* | 2 |
| *Akkermansia muciniphila* | 1 | *Bilophila wadsworthia* | 2 |
| *Akkermansia muciniphila B* | 1 | *CAG-177 sp003514385* | 2 |
| *Alistipes finegoldii* | 1 | *CAG-217 sp000436335* | 2 |
| *AM51-8 sp003478275* | 1 | *CAG-267 sp001917135* | 2 |
| *Amulumruptor sp900539915* | 1 | *CAG-568 sp000434395* | 2 |
| *Aphodousia sp900553105* | 1 | *Dialister sp900541485* | 2 |
| *Bifidobacterium bifidum* | 1 | *Duodenibacillus intestinavium* | 2 |
| *Bifidobacterium longum* | 1 | *Enterococcus faecalis* | 2 |
| *Caecibacter hominis* | 1 | *Mediterraneibacter torques* | 2 |
| *CAG-103 sp000432375* | 1 | *Odoribacter splanchnicus* | 2 |
| *CAG-115 sp003531585* | 1 | *Pelethousia gallinarum* | 2 |
| *CAG-1427 sp000436075* | 1 | *Porphyromonas uenonis* | 2 |
| *CAG-274 sp900545305* | 1 | *Porphyromonas A bennonis* | 2 |
| *CAG-41 sp900066215* | 1 | *Prevotella buccalis* | 2 |
| *Clostridium A leptum* | 1 | *Prevotella sp015074785* | 2 |
| *Collinsella sp003459245* | 1 | *Prevotella sp900548585* | 2 |
| *Collinsella sp900541695* | 1 | *Prevotella timonensis* | 2 |
| *Collinsella sp900544095* | 1 | *Ruminiclostridium E siraeum* | 2 |
| *Dialister sp000434475* | 1 | *Ruminococcus B gnavus* | 2 |
| *Duodenibacillus intestinavium* | 1 | *Varibaculum* | 2 |
| *Dysosmobacter sp001916835* | 1 | *Acetatifactor intestinalis* | 1 |
| *Eggerthella lenta* | 1 | *Akkermansia muciniphila* | 1 |
| *Fimenecus sp000432435* | 1 | *Akkermansia muciniphila B* | 1 |
| *Fimivicinus sp900544375* | 1 | *Alistipes dispar* | 1 |
| *Haemophilus D parainfluenzae* | 1 | *Alistipes putredinis* | 1 |
| *Haemophilus D sp001815355* | 1 | *Alistipes shahii* | 1 |
| *Klebsiella pneumoniae* | 1 | *Anaerofilum excrementigallinarum* | 1 |
| *Lachnospira rogosae A* | 1 | *Anaerotignum faecicola* | 1 |
| *Lachnospira sp000437735* | 1 | *Angelakisella sp900547385* | 1 |
| *Lachnospira sp900316325* | 1 | *Bacteroides clarus* | 1 |
| *Lactobacillus amylovorus* | 1 | *Bacteroides togonis* | 1 |

**Supplementary Table S4.** Cont.

| **MAGs (n=381) recovered from** | | | |
| --- | --- | --- | --- |
| **Healthy controls** | | **Outpatients recovered from acute leukemia (AL)** | |
| **Taxa** | **Frequency** | **Taxa** | **Frequency** |
| *Limisoma sp000437795* | 1 | *Bifidobacterium breve* | 1 |
| *Limosilactobacillus fermentum* | 1 | *Bifidobacterium piotii* | 1 |
| *Mesosutterella multiformis* | 1 | *CAG-103 sp000432375* | 1 |
| *Mitsuokella multacida* | 1 | *CAG-127 sp900319515* | 1 |
| *Niameybacter stercoravium* | 1 | *CAG-170 sp000432135* | 1 |
| *Odoribacter splanchnicus* | 1 | *CAG-266 sp000436095* | 1 |
| *Parasutterella excrementihominis* | 1 | *CAG-417 sp000432835* | 1 |
| *Parasutterella gallistercoris* | 1 | *CAG-83 sp000435555* | 1 |
| *PeH17 sp000435055* | 1 | *CAG-83 sp000435975* | 1 |
| *Phocaeicola vulgatus* | 1 | *CAG-882 sp003486385* | 1 |
| *Prevotella pectinovora* | 1 | *Campylobacter B ureolyticus* | 1 |
| *Prevotella sp002300055* | 1 | *Campylobacter B ureolyticus A* | 1 |
| *Prevotella sp900543975* | 1 | *Clostridium AQ innocuum* | 1 |
| *Prevotella sp900553155* | 1 | *Coprobacillus cateniformis* | 1 |
| *Ruminiclostridium E siraeum* | 1 | *Copromonas sp900066785* | 1 |
| *Scatocola faecipullorum* | 1 | *Corynebacterium pyruviciproducens* | 1 |
| *Sellimonas intestinalis* | 1 | *Desulfovibrio sp900556755* | 1 |
| *Sutterella parvirubra* | 1 | *Dialister invisus* | 1 |
| *Sutterella wadsworthensis* | 1 | *Dialister succinatiphilus* | 1 |
| *Turicibacter sanguinis* | 1 | *Duncaniella* | 1 |
| *UBA11524 sp000437595* | 1 | *Duodenibacillus intestinigallinarum* | 1 |
| *UBA1394 sp900538575* | 1 | *Enterocloster clostridioformis* | 1 |
| *Veillonella parvula A* | 1 | *Eubacterium R sp000436835* | 1 |
| *VUNA01 sp002299625* | 1 | *Faecalibacterium prausnitzii* | 1 |
| **Total** | **129** | *Faecalibacterium prausnitzii E* | 1 |
|  |  | *Faecalimonas phoceensis* | 1 |
|  |  | *Fenollaria massiliensis* | 1 |
|  |  | *Fenollaria sp900539725* | 1 |
|  |  | *Fenollaria timonensis* | 1 |
|  |  | *Haemophilus D parainfluenzae* | 1 |
|  |  | *Haemophilus D parainfluenzae J* | 1 |
|  |  | *Hungatella effluvii* | 1 |
|  |  | *Jonquetella anthropi* | 1 |
|  |  | *KA00134* | 1 |
|  |  | *Klebsiella pneumoniae* | 1 |
|  |  | *Lawsonella sp018376445* | 1 |
|  |  | *Mediterraneibacter lactaris* | 1 |
|  |  | *Merdousia sp000438015* | 1 |
|  |  | *Mesosutterella multiformis* | 1 |
|  |  | *Mitsuokella jalaludinii* | 1 |
|  |  | *Mobiluncus* | 1 |
|  |  | *Morganella morganii* | 1 |
|  |  | *Parabacteroides johnsonii* | 1 |
|  |  | *Parabacteroides merdae* | 1 |
|  |  | *Parasutterella* | 1 |
|  |  | *Parasutterella excrementihominis* | 1 |
|  |  | *Parasutterella gallistercoris* | 1 |
|  |  | *Peptoniphilus B duerdenii* | 1 |
|  |  | *Phil1 sp001940855* | 1 |
|  |  | *Phocaeicola dorei* | 1 |
|  |  | *Phocaeicola vulgatus* | 1 |
|  |  | *Porphyromonas asaccharolytica* | 1 |
|  |  | *Prevotella* | 1 |
|  |  | *Prevotella sp000436595* | 1 |
|  |  | *Prevotella stercorea* | 1 |
|  |  | *Scatocola faecipullorum* | 1 |
|  |  | *Scatomorpha* | 1 |
|  |  | *Serratia liquefaciens* | 1 |
|  |  | *SFEL01 sp004557245* | 1 |
|  |  | *SFGY01* | 1 |
|  |  | *Streptococcus anginosus* | 1 |
|  |  | *Streptococcus oralis S* | 1 |
|  |  | *Streptococcus oralis V* | 1 |
|  |  | *Sutterella merdavium* | 1 |
|  |  | *Sutterella sp900762445* | 1 |
|  |  | *UBA11524 sp000437595* | 1 |
|  |  | *UBA737 sp900549755* | 1 |
|  |  | *UMGS1071 sp900541905* | 1 |
|  |  | *Veillonella parvula A* | 1 |
|  |  | **Total** | **252** |

## Supplementary Figures

**
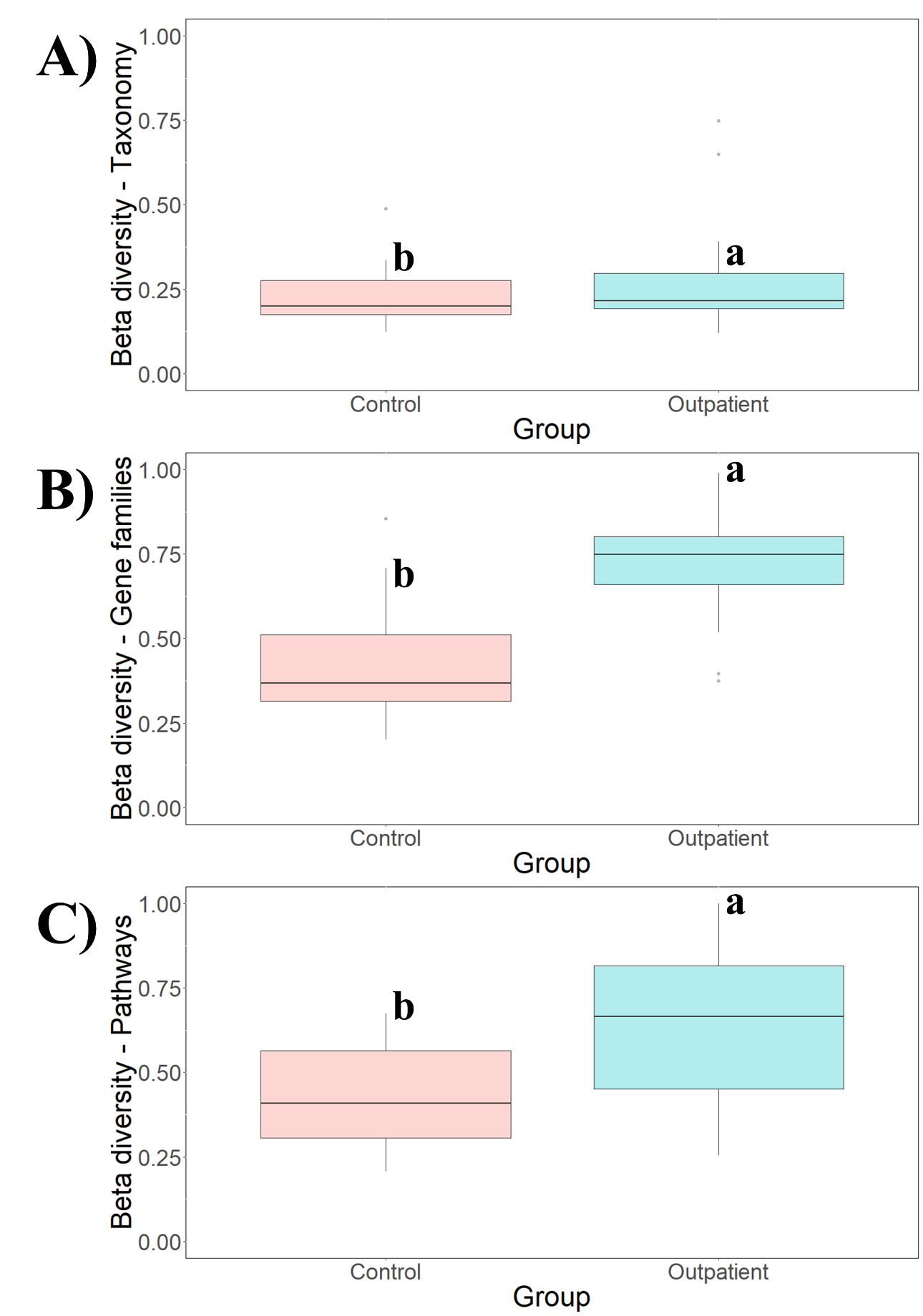
**

**Supplementary Figure S1.** Beta-diversity analysis of taxonomic profiles (**A**), gene families (**B**) and metabolic pathways (**C**) found in the microbiota of outpatients recovered from acute leukemia (AL) and healthy controls. Bray-Curtis method was selected for the calculation. ^a,b^ Statistically significant differences (*p* < 0.05) between groups determined by PERMANOVA.


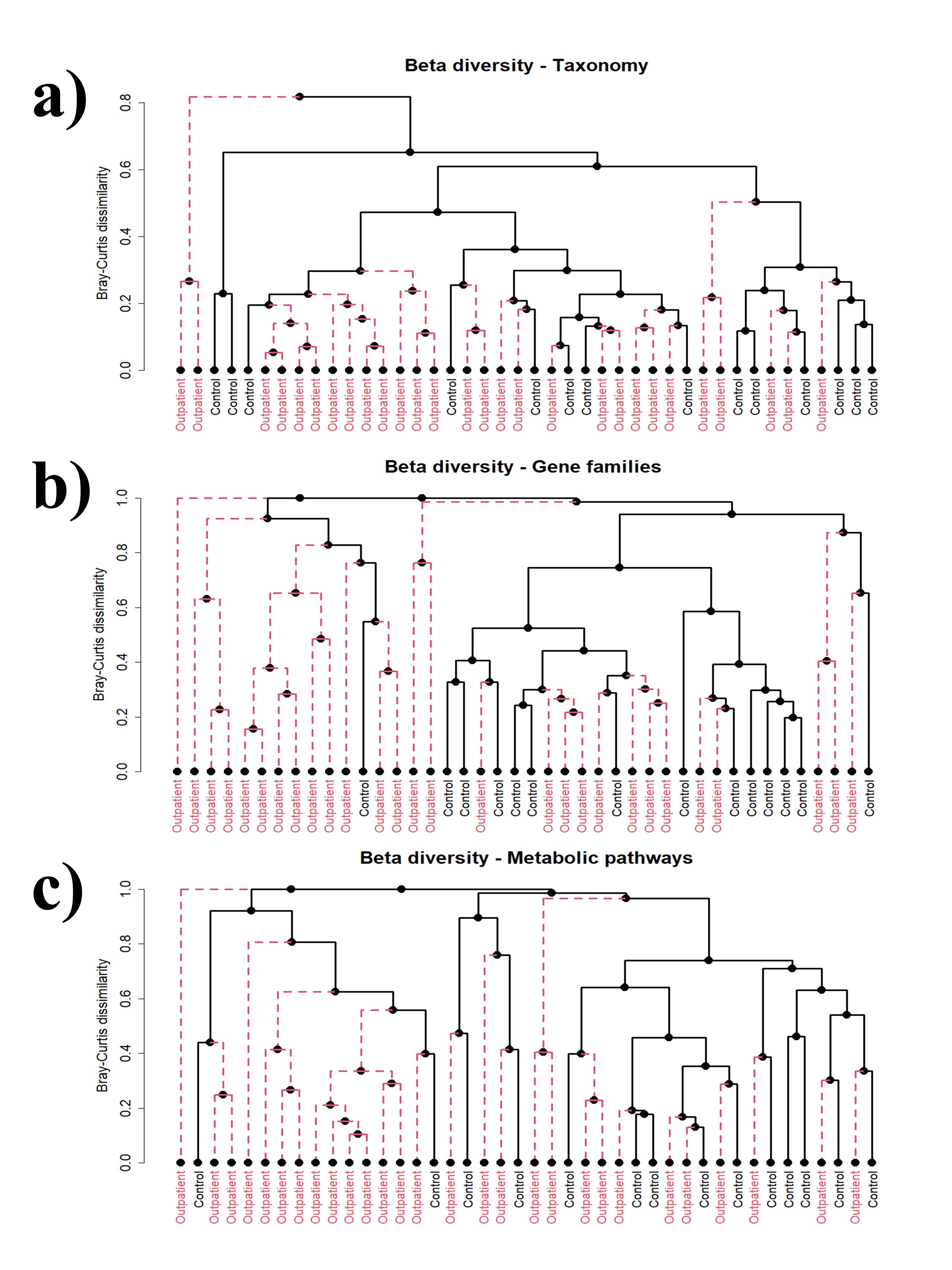


**Supplementary Figure S2.** Clustering of analysis of taxonomic profiles (**A**), gene families (**B**) and metabolic pathways (**C**) found in the microbiota of outpatients recovered from acute leukemia (AL) and healthy controls. Bray-Curtis dissimilarity method was selected for the calculation. As it can be seen, few samples corresponding to the same group were clustered together. These results highlight the role of interindividual variability.


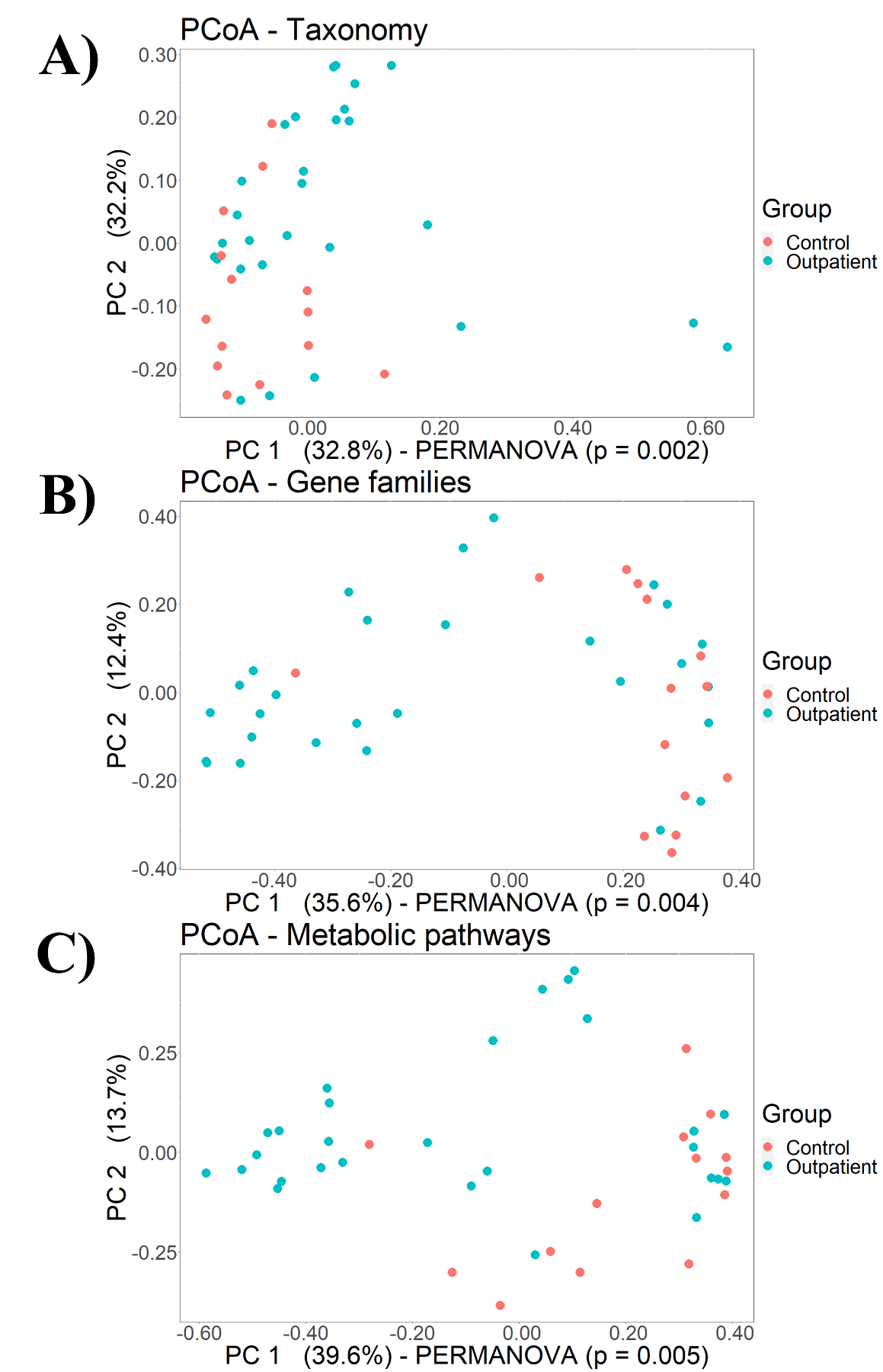


**Supplementary Figure S3.** Principal coordinates analysis (**PCoA**) of taxonomic profiles (**A**), gene families (**B**) and metabolic pathways (**C**) found in the microbiota of outpatients recovered from acute leukemia (AL) and healthy controls. **PC:** principal coordinate. The percentage of variance explained by each PC is indicated in the axis. Statistically significant differences (*p* < 0.05) between groups determined by PERMANOVA are illustrated.

**
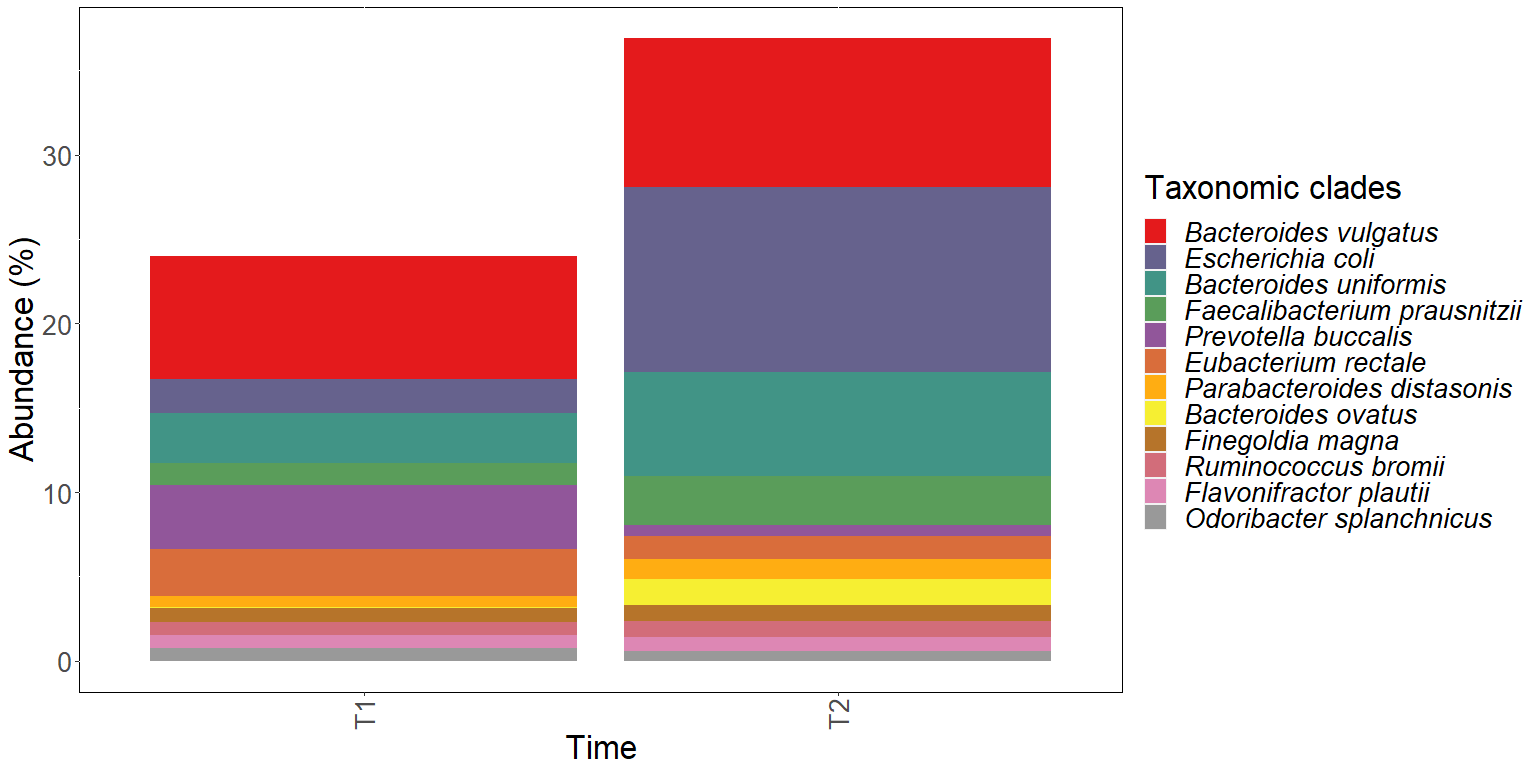
**

**Supplementary Figure S4.** Most abundant taxa found in the microbiota of early and delayed recovery (T1 and T2, respectively) of outpatients recovered from acute leukemia (AL). These taxa constitute the core microbiota of individuals. Data are expressed as abundance percentages (%).
